# Hypoxia-induced alternative splicing: the 11th Hallmark of Cancer

**DOI:** 10.1186/s13046-020-01616-9

**Published:** 2020-06-15

**Authors:** Antonietta Rosella Farina, Lucia Cappabianca, Michela Sebastiano, Veronica Zelli, Stefano Guadagni, Andrew Reay Mackay

**Affiliations:** Department of Applied Clinical and Biotechnological Sciences, University of L’Aquila, 67100 L’Aquila, Italy

**Keywords:** Hypoxia, Alternative splicing, Cancer hallmarks

## Abstract

Hypoxia-induced alternative splicing is a potent driving force in tumour pathogenesis and progression. In this review, we update currents concepts of hypoxia-induced alternative splicing and how it influences tumour biology. Following brief descriptions of tumour-associated hypoxia and the pre-mRNA splicing process, we review the many ways hypoxia regulates alternative splicing and how hypoxia-induced alternative splicing impacts each individual hallmark of cancer. Hypoxia-induced alternative splicing integrates chemical and cellular tumour microenvironments, underpins continuous adaptation of the tumour cellular microenvironment responsible for metastatic progression and plays clear roles in oncogene activation and autonomous tumour growth, tumor suppressor inactivation, tumour cell immortalization, angiogenesis, tumour cell evasion of programmed cell death and the anti-tumour immune response, a tumour-promoting inflammatory response, adaptive metabolic re-programming, epithelial to mesenchymal transition, invasion and genetic instability, all of which combine to promote metastatic disease. The impressive number of hypoxia-induced alternative spliced protein isoforms that characterize tumour progression, classifies hypoxia-induced alternative splicing as the 11th hallmark of cancer, and offers a fertile source of potential diagnostic/prognostic markers and therapeutic targets.

## Background

Tumour chemical and cellular microenvironments interact continually to select survival-adapted tumour cell and tumour-associated normal cell populations, and underpins both metastatic progression and therapeutic resistance. The tumour cellular microenvironment is comprised of “normal” (vascular, stromal and inflammatory cells) and neoplastic components that co-exist within a poorly defined and poorly organized extracellular matrix, characterized by heterogeneous niches created by a highly abnormal vasculature and episodes of microenvironmental hypoxic, nutrient, metabolic and redox stress, which elicit cellular hypoxic, nutrient, oxidative and metabolic stress responses. Tumour hypoxia promotes glycolytic metabolic adaptation by tumour cellular components, combined with oncogene-promoted metabolic changes, result in the malignant tumour-associated “Warburg” metabo-type [1–3]. The metabo-type, furthermore, promotes an acidic reducing tumour microenvironment, which together with tumour hypoxia, acts as potent driving forces for survival adaptation [4, 5], selecting “normal” and neoplastic tumour cellular components that exhibit increased resistance to programmed cell-death, a pro-angiogenic phenotype, sustained metabolic glycolytic reprogramming, progressive epithelial/mesenchymal (EMT) and stem cell-like de-differentiation, enhanced motile, invasive, scattering and metastatic behaviour, increased genetic instability and enhanced therapeutic resistance [5–13].

## Tumour hypoxia

Tumour-hypoxia results when tumour cellular components are deprived of oxygen and occurs during all phases of tumour progression, from early initiation through clonal expansion to metastatic progression [14]. Solid tumours are characterized by heterogenous hypoxic areas adjacent to near normoxic regions and exhibit [pO_2_] concentrations ≤2.5 mmHg, significantly below those of normal vascular tissues, as a result of an imbalance between oxygen consumption and supply, e.g. [pO_2_] of 10–16 mmHg in cervical tumour tissues is significantly lower than the [pO_2_] 40–42 mmHg of normal cervical tissues [9, 10, 15].

**Fig. 1:**
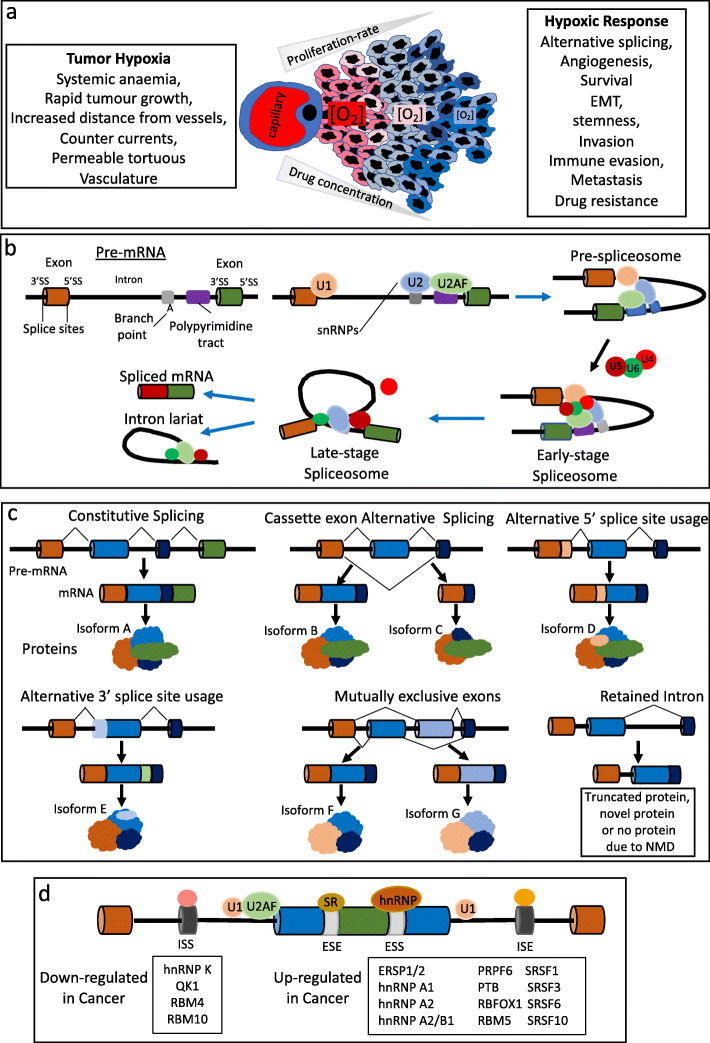
Tumour hypoxia, constitutive and alternative Pre-mRNA splicing. Schematic representations of: **a** tumour hypoxia, the mechanisms involved in promoting the hypoxic tumour microenvironment and resulting cellular tumour promoting hypoxic response, including hypoxia-induced alternative splicing; **b** splice site, intron and exon architecture and interaction with splicing factors and spliceosome components that select splice sites and eliminate intron sequences via the formation of a lariat structure, followed by the splicing together of exons; **c** constitutive pre-mRNA splicing and alternative splicing by cassette exons skipping, alternative 5′ splice site use, alternative 3′ splice site use, the use of mutually exclusive exons and by retaining introns; **d** ESE, ESS, ISE and ISS splice elements plus splicing factors down-regulated or up-regulated in cancer

Tumour hypoxia arises from a variety of mechanisms. Tumour perfusion-hypoxia is caused by an abnormal disorganized tumour vasculature, characterized by structural, functional and cellular abnormalities and inadequate blood flow, resulting in transient ischemic episodes of varying duration caused by blockage and/or flow stasis. Tumour diffusion-hypoxia is caused by O_2_ diffusion distances > 70 μm between tumour tissues and blood vessels, and blood flow countercurrents within the tumour microvascular. Tumour anemic hypoxia is caused by reduced O_2_ transport capacity resulting from the tumour itself or by systemic anemia caused by chemotherapy (Fig. 1a). In general, tumour-hypoxia is independent of tumour size, stage, histopathological type and grade, and also independent of patient age, parity, menopausal status and smoking habits [6, 7, 16].

## Pre-mRNA splicing and alternative splicing

Pre-mRNA splicing represents the process whereby non-coding intronic sequences within a gene are coordinately excised from pre-mRNA transcripts, and coding exons are ligated together to form a single mature protein encoding mRNA molecule. This maturation process occurs within nuclear speckles, which are sites of active transcription. Alternative splicing represents the exclusion or inclusion of different exons and/or intron sequences within the mature mRNA sequence [16, 17]. As genes numbers stopped increasing during evolution, alternative splicing became the main source of protein complexity, and functional diversity. The current alternative splice record is held by the *Drosophila DISCAM* gene, which is expressed as 38,000 individual splice variants, which represent more than the entire number of *Drosophila* genes [18]. In humans, alternative splicing accounts for ≈ 100,000 different proteins, is largely responsible for proteomic complexity that cannot be explained by gene numbers alone and is tightly regulated in order to provide sufficient adaptive flexibility to gene expression, whilst limiting the potential for chaos [19, 20].

Splicing initiates with spliceosome recruitment to the 5′ exon-intron splice junction and subsequent phosphodiester bond cleavage at the 5′ splice site, in a process involving a branch point adenosine and formation of an intermediate lariat structure, subsequently liberated by phosphodiester bond cleavage at the 3′ splice site exon-intron junction, which also depends upon a free 5′ exon hydroxyl group. Following intron splicing, exons are ligated together to form an in-frame mature protein encoding mRNA sequence (Fig. 1b). Alternative splicing is regulated by many factors, including enhancer and/or silencer *cis-elements* located within exons and/or introns that bind heterogeneous RNA binding (hnRNPs) or serine-arginine-rich (SR) *trans-acting* proteins, relative splice-site strengths, the localization of splice enhancing and/or silencing *cis-elements*, pre-mRNA secondary structure, the transcriptional elongation rate, the lengths of exons and introns, and the presence of modified RNA nucleotides (Fig. 1c and d) [21–25].

The 5′ splice site is composed of 9 nucleotides, demarcates the exon-intron boundary and recruits U1 snRNP. The 3′ splice site contains an AG dinucleotide that delineates the exon-intron boundary and contains an upstream polypyrimidine tract, responsible for recruiting U2AF heterodimers, the U2AF65 component of which binds the pyrimidine tract and the U2AF35 subunit binds the AG dinucleotide, facilitating U2 snRNP recruitment to the intronic branch point. Alterations in these interactions regulate alternative splicing and result in either exon cassette inclusion or skipping, intron retention, mutually exclusive exon use, alternative first and last exon use, alternative 5′ and 3′ splice site use or the selection of alternative 5′ and 3′ untranslated regions (UTRs). Splice site strength is calculated by maximum entropy principle and dictates spliceosome component recruitment and assembly. The 5′ and 3′ splice sites play equal roles in cassette exon inclusion and the sum of 5′ and 3′ splice site scores predicts exon inclusion. Pre-mRNA secondary structure also regulates alternative splicing, as spliceosome components and regulators bind single stranded RNA and can be masked by secondary structure. Splicing can also be influenced by protein interaction (e.g. hnRNPA1 promotes distal 5′ spice site activation by looping out an internal exon), which results in ≈ 4% of alternative splicing events. Regulation of alternative splicing by *cis-elements* depends upon recruitment of *trans-acting* hnRNPs and SR splicing factors that are required for spliceosome assembly. *Cis-element* localization is critical for this process and may act either as an exon splice enhancer (ESE), exon splicing silencer (ESS), intron splicing enhancer (ISE) or intron splicing silencer (ISS). ESEs recruit SR proteins to exons and localize spliceosome components adjacent to the intron via protein-protein interactions, whereas ESSs recruit hnRNPs to pre-mRNAs to repress exon inclusion. In general, SR proteins bound to exons upstream of the 5′ splice site activate splicing but repress splicing when bound to introns downstream of 5′ splice sites, with alternative splicing promoted by alterations in splice site *trans-acting* SR and hnRNP protein expression. RNA polymerase II elongation rates, which are regulated by hypoxia, also regulate alternative splicing, with faster rates facilitate exon skipping, and slower rates facilitating sub-optimal splice-site recognition and RNA secondary structure formation (e.g. in fibronectin ED1 exon inclusion or exclusion) [26, 27]. With respect to exon and intron size, large exons (> 500 nucleotides) flanked by large introns (> 500 nucleotides) are more likely to be skipped and recognized when flanked by short exons (< 500 nucleotides). In contrast, short exons (< 500 nucleotides) are recognized when flanked by large introns (> 500 nucleotides) [28, 29].

Post-transcriptionally modified nucleotides in pre-mRNAs and snRNAs also influence spliceosome recruitment and promote alternative splicing. 2′-O-methyl, pseudo-uridine and trimethylated guanosine cap (m3G) modifications in U2 SnRNAs are critical for splicing reactions and nuclear U-snRNP importation, post-transcriptional m6A modifications in pre-mRNAs influence secondary structure, altering single-strand RNAs and RNA binding motif accessibility, and adenosine deaminase conversion of adenosine to inosine creates novel splice sites by converting AA dinucleotides to AI dinucleotides that promote alternative splicing [30]. Alternative splicing occurs in ≈ 86–88% of human genes. It is a highly complicated process that is tightly regulated under physiological conditions and responsible for the transcriptome diversity required for all aspects of physiological cell behaviour (Fig. 1b, c and d).

## Hypoxia-induced gene expression and alternative splicing

The response to hypoxia includes a series of adaptation mechanisms that promote cell survival. At the systemic level, the carotid body within the carotid artery senses decreased O_2_ levels and stimulates breathing and cardiovascular output [31]. This response involves calcium and voltage activated K (BK) channels expressed in the carotid body and also by neuroepithelia, the α subunits of which are sensitive to alternative splicing, with hypoxia inducing inclusion of the stress-regulated exon STREX to confer sensitivity to hypoxia in a tissue specific pattern, providing a tissue-specific mechanism to control cellular responses to hypoxia [32]. Cellular molecular oxygenation sensing depends also upon oxygen-dependent oxygenases, comprised of a family of 2-oxoglutarate-dependent oxygenase, including the hypoxia-inducible factor (HIF) oxygen-dependent prolyl-hydroxylase PHD [33]. Hypoxia inhibits PHD activity resulting in the accumulation, stabilization and activation of HIF transcription factors, that promote HIF-target gene expression, alternative splicing of HIF-target and non-HIF target genes and also induce 4E-BP1 phosphorylation-dependent inhibition of capped non-HIF target gene mRNA translation, also inhibited by the hypoxia-induced RNA binding protein EVLAV1 (HuR) that regulates the expression of translation initiating factor 4E nuclear import factor 1 (Eif4enif1) [34–40].

Under normoxic conditions, proline hydroxylated HIF1α is targeted for proteasomal degradation by the von-Hippel Lindau tumour suppressor (pVHL), complexed with elongin B, elongin C, Cullin2 and Rbx1 (33). This mechanism is inactivated by hypoxia, resulting in HIFα dissociation and stabilization, nuclear translocation and formation of HIF α/β heterodimers, composed of one of three α subunits (HIF1α, HIF2α and HIF3α) and one of two β subunits (HIF-β and ARNT2), leading to HIF-binding to hypoxia responsive elements (HREs) in gene promoters and transcription of an impressive number of HIF-target genes, involved in metabolic adaptation, angiogenesis, survival, cellular motility, staminality and metastatic progression [13, 41–44]. This response also involves alternative splicing of peptidyl prolyl isomerase-1 (Pin1), which binds and stabilizes HIF1α [45], by repressing long non-coding (Lnc) RNA PIN1-v2 alternative splice variant that inhibits HIF1α transcription, implicating the hypoxia-regulated alternative Pin1 splice equilibrium in hypoxia-induced, HIF-1-dependent gene expression [46]. Hypoxia also activates p50/p65 NF-κB transcription factor that is also negatively regulated by PHD-mediated proline hydroxylation [47], promotes CREB phosphorylation-dependent transcription [48] and enhances NF-E2-related factor 2 (Nrf2) [49], STAT [50] and c-Myc transcriptional activity, confirming regulation of both HIF-target and non-HIF-target gene transcription.

Hypoxia-induced alternative splicing is critical for adaptation of both normal and tumour cellular microenvironments and is central to one of the most important functions of the normal and tumour hypoxic responses, angiogenesis, responsible for vascularizing hypoxic tissues [51]. The neovascularization of hypoxic tissues is achieved by lowering the ratio of angiogenesis inhibitors to angiogenesis promoters and depends upon hypoxia-induced, HIF-dependent, alternative splicing that promotes a pro-angiogenic VEGFA_165a_ alternative splice equilibrium, at the expense of the anti-angiogenic VEGFA_165b_ isoform (see below). Hypoxia also regulates HIF-1α splicing during angiogenesis and promotes expression of the angiogenesis inhibitory alternatively spliced HIF-3α IPAS isoform, that binds HIF1α but not HIF-β to inhibit HIF-1-mediated transcription, up-regulates alternative HIF-3α4 splicing to suppresses HIF-dependent transcription and also induces the expression of a dominant negative exon 11 and 12 skipped HIF-1α516 isoform, providing negative feedback loops that also regulate metabolism, confirming a high degree of complexity in hypoxia-regulated alternative splicing in angiogenesis [52, 53].

## Hypoxia-induced alternative splicing in cancer

Hypoxia induces alternative splicing in normal and neoplastic tumour components. In human endothelial cells hypoxia been shown to induce 342 alternative splicing events [54], in liver cancer cells induces 3059 alternative splicing events in 2005 genes, contributing to dedifferentiation and genome instability [55], and in breast cancer cells ≈2000 alternative splicing events, with estimated alternative splice rates of ≈1.78 events per HIF-target gene and ≈1.53 events per non-HIF-target gene, distributed relatively evenly between exon cassettes inclusion and exclusion reported in breast cancer, hepatocellular carcinoma, neuroblastoma and head and neck squamous carcinoma cells [56]. With respect to HIF-target genes, the majority of hypoxia-induced alternative splicing events involve genes that regulate oxy-reductase activity, glycolysis, glucose uptake, ATP-binding, protein kinase activity, pleckstrin homology, rho signaling, cytoskeletal organisation and cell death and, in general, favor expression of full length exon-included over exon-skipped isoforms, whereas hypoxia-induced exon-excluded isoforms are predominant in non-HIF target genes [36]. Deep sequencing in 16 different cancer types, including breast, colon, head and neck and lung cancers has also identified > 1000 hypoxia-induced alternatively spliced transcripts with 23 different alternative splice protein isoforms, associated with altered expression of RNA splicing factors SF1, SRSF1, SRSF3 and SRSF7, SF3 gene repression and expression of translation initiating E1F2B family members E1F5 and EIF6, and has identified 1103 late exon, intron retention and tandem 3′ TRS alternative splice events in 819 unique genes involved in protein translation, mitochondrial and ER protein degradation, metabolism, programmed cell death [57].

The effects of hypoxia on the general splicing machinery, include de-regulation of SRSF1, SRSF2, SRSF3, SAM68, HuR, hnRNPA1, hnRNPM, PRPF40B and RBM4 splice factor expression, activation and increased expression of the SR protein kinases Cdc2-like kinase-1 (CLK1) and SRPK1, that promote SR splice factor hyper-phosphorylation and activity, alter splice factor intracellular localization, and capacity to interact with other proteins and pre-mRNAs, resulting in hypoxia-adapted gene transcription and promotion of tumour progression [58–63]. Amongst splice factors, hypoxia also induces alternative splicing of the ubiquitous splicing factor YT521 (YTHDC1), switching expression to two non-coding YT521 variants 2 and 3 mRNAs, functionally coupled to nonsense mediated decay, that impact the splicing of cancer-associated BRCA2 and PGR [64]. Hypoxia also significantly alters the expression of miRNAs involved in splicing and induces the expression of master lncRNA regulators of alternative splicing MALAT1, HOTAIR and LUCAT [65–71].

Hypoxia sensitive signal transduction pathways also regulate alternative splicing, resulting in tumour promoting VEGF, FGF, HGF and TGFβ signaling, ligand-independent EGFR signaling, myogenic to mitogenic conversion of insulin growth factor signaling and also specify signaling pathways use [72–76]. Signaling pathways that promote alternative splicing include: KRas promotion of PTBP1 splicing factor, Rho GTPase Rac1b, endocytic adapter NUMB and pyruvate kinase PKM2 alternative splicing; ERK promotion of splice factor phosphorylation, cancer progression-promoting CD44 exon V5 alternative splicing, fibronectin EDA exon inclusion, FAS exon 6 exclusion via SPF45 phosphorylation and SRSF1 splice factor repression via intron retention [77]; BRAF promotion of pre-mRNA processing factor phosphorylation, nucleo-cytoplasmic transport and localization, Bcl-xL alternative splicing and repression of dominant negative A-Raf expression via hnRNPA2-dependent alternative splicing; PI3K/Akt promotion of SRSF1 and SRSF7 phosphorylation, SRPK1 and SRPK2 autophosphorylation, fibronectin ED1 exon inclusion, inhibitory caspase 9 exon 3–6 exclusion, SRSF1-dependent KLF6 SV1 and SRSF5-dependent PKCβII alternative splicing, SR import into nuclear speckles and mTORC1/S6k1-induced lipogenesis-related gene alternative splicing; Wnt promotion of SRSF3 expression, Rac1b alternative splicing, SRPK1 and SRSF1-dependent SLC39A14 alternative 4A and 4B exon splicing; cAMP promotion of cytoplasmic PTBP1 accumulation; WT-1 repression of SRPK1 expression and promotion of pro-angiogenic VEGFA alternative splicing; casein kinase 2 (CK2) activation of SRPK1, and calcium promotion of CaMKIV-dependent hnRNPL phosphorylation and binding to RNA CARRE motifs that regulate gene-specific alternative splicing, all of which are influenced by tumour hypoxia [78].

Hypoxia also influences alternative splicing indirectly by promoting the formation of cytosolic Stress Granules, containing stalled translation pre-initiation complexes comprised of mRNAs, translation initiating factors, ribosomal subunits and RNA binding proteins, and closely related GW/P bodies that contain mRNAs, mRNA transport and modification factors, mRNA decay enzymes, translational repressor proteins. Stress granules store mRNAs, act as miRNA-mediated gene-silencing centres and contribute to cancer aggressiveness by regulating cell-death, tumourigenesis, therapeutic resistance and metastatic capacity. Stress granules regulate hypoxia-induced alternative splicing [79–82] by accumulating SRSF splicing factors and splice regulating CELF proteins that promote non-sense-mediated mRNA decay, and through stress-induced maturation of miRNAs that regulate splicing, such as miR-133 which targets hnRNP1/PTBP1 splicing factor. Stress granules also accumulate TDP43 splice factor, a component of Dicer complexes that drive stress-induced granule dynamics and miRNA biogenesis [65, 83, 84] (Fig. 4). Hypoxia is, therefore, a master regulator of stress-granule-associated microRNA biogenesis and activity, further influencing alternative splicing at the post-transcriptional level [85]. Hypoxia-induced alternative splicing is, therefore, highly complex, fundamental for normal physiological development, cellular differentiation and adaptive cellular responses and is subverted within the tumour context to promote metastatic progression and therapeutic resistance [86].

In the following sections, we review current concepts of the many cancer-associated hypoxia-regulated alternative splicing events that regulated tumour behaviour, organized with respect to the 10 hallmarks of cancer and the prospects for therapeutic intervention.

## Hypoxia-induced alternative splicing in autonomous neoplastic growth (hallmark 1)

Tumour initiation is determined by a combination of oncogene activation and tumour suppressor inactivation, resulting in the acquisition of autonomous neoplastic growth that is promoted either by autocrine growth factor activity caused by coincidental tumour cell growth factor and growth factor receptor expression or by proliferation-promoting oncogenes damage-activated by oncoviruses, gene amplification, mutation, chromosomal translocation or alternative/aberrant pre-mRNA splicing. Rapid autonomous neoplastic growth results in tissue hypoxia at O_2_ diffusion distances > 70 μm, resulting in a pro-angiogenic hypoxic responses, cell-death and an acute inflammatory response, also required for tumour angiogenesis and clonal expansion. During this phase, tumour hypoxia-induced alternative splicing influences oncogenic activity both directly and indirectly, helping to promote and maintain tumour autonomous growth potential (Fig. 1a) [9–15].

**Fig. 2:**
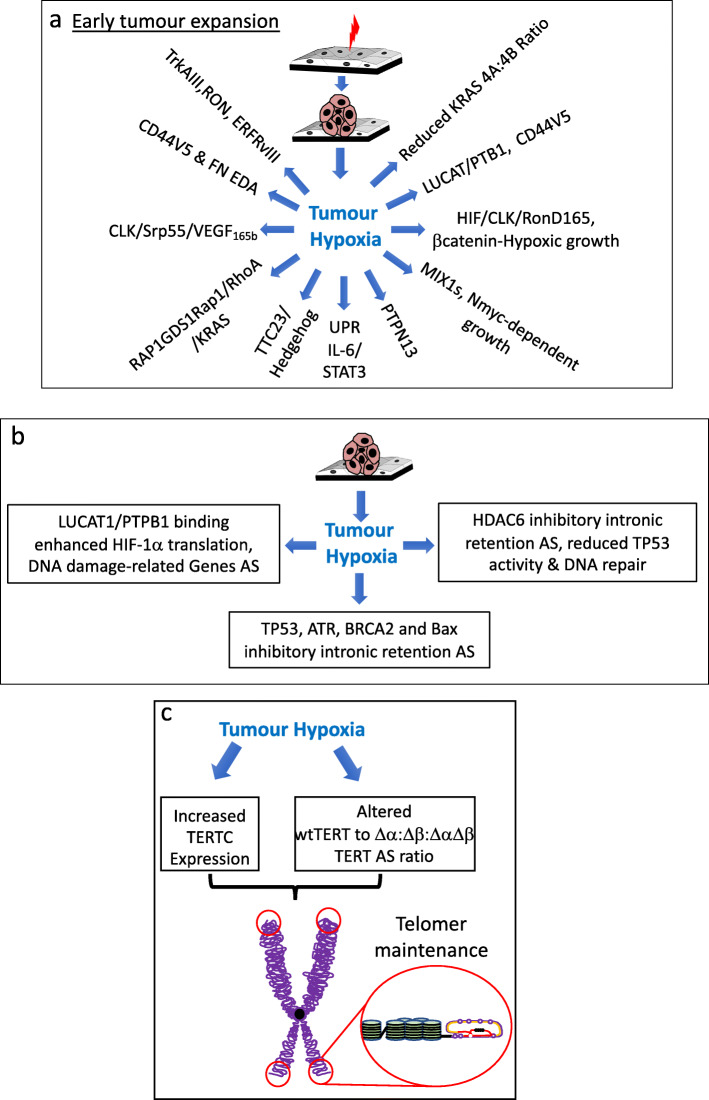
Tumour hypoxia-induced alternative splicing, autonomous growth, tumour suppressor inactivation and immortalization. Schematic representations of the numerous roles played by tumour hypoxia-induced alternative splicing (AS) in: **a** autonomous neoplastic growth; **b** tumour suppressor inactivation and **c** tumour cell immortalization

Receptor tyrosine kinase proto-oncogenes [87] that interact with the hypoxic tumour microenvironment [88], resulting in oncogenic activation, include the neurotrophin tropomyosin-related tyrosine kinase receptor TrkA that exhibits hypoxia-induced oncogenic alternative TrkAIII splicing in human neuroblastoma, pheochromocytoma, leukemia and medullary thyroid cancer cells. TrkAIII is expressed by advanced stage primary human neuroblastomas, glioblastomas, melanomas and Merkel cell carcinomas, is characterized by cassette exon 6, 7 and 9 skipping, exhibits constitutive activation, transforms NIH3T3 cells, exhibits oncogenic activity in neuroblastoma models and prevents neural-related progenitor cell death induced by the development-regulated *NF-YA* alternative splice variant NF-YA*x*, expressed during mouse developmental stages associated with neuroblast culling and neuroblastoma suppression, suggesting potential roles in neuroblastoma initiation and hypoxia-dependent progression [89–92]. Hypoxia also promotes aberrant/alternative splicing of the epithelial growth factor receptor EGFR, resulting in expression of the constitutively active, exon 2–7 skipped EGFRvIII (ΔEx 2–7) isoform, a proliferation promoting driver-oncogene in several tumour-types, including glioblastoma multiforme [93–95], and also induces pro-proliferation Erb4 signaling in mammary epithelial cells [96]. Hypoxia reduces the KRAS 4A to 4B (exon 4a skipped) alternative splice ratio, helping to explain predominant mutation-activated KRAS4B splice variant oncogene expression in colon tumours and cancer stem cells [36, 97, 98], and induces predominant short form MXIs alternative splicing reducing MIX1 antagonism of Nmyc-dependent proliferation of relevance to aggressive autonomous Nmyc amplified neuroblastoma growth [57]. In prostate cancer cells, hypoxia induces non-catalytic alternative splicing of the tyrosine-protein phosphatase PTPN13, augmenting tyrosine kinase-dependent signaling and proliferation, induces alternative *TTC23* splicing involved in hedgehog signaling and promotes alternative RAP1GDS1 splicing, enhancing GDP/GTP exchange reactions in Rap1a and 1b, RhoA and B and KRas G-proteins, promoting autonomous growth (Fig. 2a) [99].

In colorectal cancer cells, hypoxia augments the expression and activity of hnRNPA1, Srp55, SF/ASF, Tra-2 beta YB-1 and Sam68 splicing factors, resulting in proliferation-promoting alternative CD44v5 and fibronectin EDA exon splicing; promotes LUCAT1 lncRNA expression and LUCAT/PTBP1 complexing, inducing 63 alternative splicing events (36 skipped and 27 retained exon events) in cell growth, cell cycle and G2/M checkpoint genes that augment tumour cell proliferation and colony formation [100], and induces alternative CD44v5 splicing, resulting in a novel cytokine and growth factor receptor isoform that promotes autonomous growth [77]. In breast cancer cells, hypoxia induces alternative *APP* splicing linked to breast cancer cell proliferation and tumorigenicity [101] and in non-small cell lung cancer cells, promotes Clk1-dependent Srp55 splicing factor phosphorylation, resulting in alternative VEGFA_165b_ splicing and autonomous growth of VEGFR2 and neuropilin-1 receptor expressing tumour cells [102, 103]. In pancreatic cancer, tumour growth under hypoxic conditions has also been attributed to hypoxia-induced alternative splicing of tissue factor, resulting in as-TF expression, which activates carbonic anhydrase IX implicated in late-stage pancreatic cancer growth under hypoxic conditions (Fig. 2a) [104].

Hypoxia-induced alternative splicing also regulates the activity of the HIF-1-target proto-oncogene RON, an epithelial cell-specific c-MET family tyrosine kinase receptor that binds macrophage specific protein (MSP). RON exhibits hypoxia-induced oncogenic alternative splicing in breast, lung, liver, kidney, bladder, ovarian, colon, pancreatic, gastric and prostate carcinomas and many cancer cell lines and is composed of heterodimers of an extracellular 40kda α chain and 150 kDa β chain that contains extracellular, transmembrane and intracellular tyrosine kinase domains, derived from the same immature pre-protein. RON activation results in intracellular phosphorylation-dependent, SH2-domain adapter protein binding to the β-chain, resulting in IP3K/Akt and MAPK signaling. Alternative *RON* splicing is complex and results in RONΔ170, Δ165, Δ160, Δ155, Δ110, Δ90 and Δi55 isoforms, several of which exhibit constitutive oncogenic activation, differences in localization, opposing functions and associate with tumour progression and disease stage. Hypoxia induces oncogenic alternative RONΔ165 splicing by promoting CLK1-mediated, SF2/ASF splice factor phosphorylation-dependent binding to an EES adjacent to an ESS *cis-element,* resulting in exon 11 skipping. Constitutive RONΔ165 activation promotes RON and β-catenin nuclear translocation, inducing cJun expression and promoting proliferation [99, 104–110]. Furthermore, increased nuclear β-catenin levels, induces TCF4 transcription factor activation, β-catenin/TCF4 complexing and the induction of cMyc, Cyclin D and c-Jun β-catenin/TCF4 target gene expression in gastric cancer cells, promoting proliferation. In addition, complexes between constitutively active RON splice variants and β-catenin also interact with HIF1α to regulate HIF-1-dependent transcription and tumour cell proliferation under hypoxic conditions, confirming a close relationship between hypoxia-induced alternative RON splicing, β-catenin and HIF-target genes in the regulation of autonomous tumour cell growth and tumour progression (Fig. 2a) [99, 105–111].

In addition to direct oncogene activation, hypoxia-induced alternative splicing also indirectly promotes autonomous growth by activating the unfolded protein response (UPR) in response to ER stress resulting from the accumulation of damaged and misfolded proteins [112, 113]. The UPR is mediated by ER ATF6, PERK and Ire1α proteins, the activation of which results in transient attenuation of protein synthesis, increased protein trafficking through the ER, augmented protein-folding capacity, protein degradation through ERAD and autophagy. Hypoxia-induced Ire1α activation results in unconventional alternative splicing of a 26 nucleotide intron from the transcription factor XBP1u, resulting in expression of the frame shift XBP1s isoform, that contains a novel transcriptional activating domain and exhibits transcriptional activity. Both XBP1u and XBP1s isoforms contain leucine zipper DNA binding domains and interact to regulate nuclear translocation and transcription, and XBP1s cooperates with HIF-1α to promote cell survival [114]. XBP1s binds CRE elements in proliferation, survival and protein-overload response genes, activates NF-κB, AP-1 and Myc oncogenic pathways, up-regulates the expression of 162 proliferation, protein folding and survival genes in human breast cancer cells, augments CD4K, c-Myc and Cyclin D expression to promote proliferation, complexes with and augments the transcriptional activity of c-Myc, promotes PI3K/mTOR-dependent osteosarcoma growth, maintains the autonomous growth potential of multiple myeloma cells [115], and promotes autocrine/paracrine STAT3-dependent growth of hepatocellular carcinoma cells [116] (Fig. 2a).

## Hypoxia-induced alternative splicing in tumour suppressor inactivation (hallmark 2)

Tumourigenesis also depends upon tumour suppressor inactivation to overcome oncogene-induced senescence. Under normal circumstances, tumour suppressors, activated by cellular damage and by activated oncogenes, inhibit proliferation by activating cell-cycle checkpoints and promote temporary survival, during which attempts are made to eliminate or repair damaged molecules and, if appropriate, induce programmed cell death. In addition, tumour suppressors also help maintain cellular differentiation, intercellular adhesive interactions and contact-dependent growth inhibition [117].

Hypoxia induces alternative intron-retention splicing repressing the expression of TGFβ1, responsible for indirect retinoblastoma protein-dependent activation of the G1/S cell-cycle checkpoint [57]. In breast cancer cells, hypoxia induces splice-dependent intron-retention nonsense mediated decay (NMD) of TP53, ATR, BRCA2 and Bax tumour suppressor mRNAs, de-regulating the DNA damage response, TP53 involvement in cell cycle arrest and BAX-dependent apoptosis, TP53 expression and function, and represses TP53-target and related gene expression [56]. In colon cancer cells, hypoxia also reduces TP53 function by promoting inhibitory alternative *HDAC6* intron-retention splicing, de-regulating the unfolded protein response (UPR), protein aggregate processing, altering the cell response to cytotoxic stress, reducing HDAC6-dependent TP53 binding protein-1 expression, repressing expression of the p53 target gene *P21/Waf1* cell cycle inhibitor and impairing recognition of H4K20me2 and H2AK15ub histone marks induced by DNA double strand breaks and DNA repair [118]. Whether hypoxia also promotes dominant negative inhibitory alternative delta-N p53, p63 and p73 splicing [119–121], remains to be confirmed.

Hypoxia also induces the expression of LUCAT1 lncRNA in cancer cells, which binds PTBP1 splicing factor resulting in alternative splicing inactivation of DNA damage-related tumour suppressors. Furthermore, PTBP1 binds the 5′ UTR internal ribosome entry site in HIF1α mRNA, enhancing HIF1α translation, and accounts for 40–50% of hypoxia-stabilized HIF1α levels [122], increasing the influence of hypoxia-induced LUCAT1/PTBP1 complexing on tumour suppressor inactivation through alternative splicing [123] (Fig. 2b).

## Hypoxia-induced alternative splicing in replicative immortality (hallmark 3)

Tumour cells exhibit replication immortality and do not respect the “Hayflick” replication limit imposed on normal cells by telomer loss [124]. Tumour cells overcome telomer shortening by de novo telomer synthesis, which depends upon the maintenance of telomerase expression, activity, and active forms of TERT telomerase reverse transcriptase. TERT/TERTC interaction align telomerase to chromosome ends, resulting in telomer DNA addition. Hypoxia increases TERT and TERTC expression and regulates TERT alternative splicing, altering the ratio between fully spliced wild type TERT and Δα, Δβ and ΔαΔβ TERT isoforms. In ovarian cancer cells, hypoxia induces predominant active wild type-TERT isoform expression, increasing both TERT and telomerase activity maintaining telomer length [125]. In stem cells, hypoxia promotes TERT Δα and Δβ alternative splicing and nuclear localization, in a stem cell maintenance mechanism, the steric inhibition of which results in differentiation, suggesting that hypoxia promotion of tumour cell alternative TERT Δα and Δβ splicing may not only maintain telomer length and promote immortality but also cancer staminality [126–130] (Fig. 2c).

## Hypoxia-induced alternative splicing in tumour angiogenesis (hallmark 4)

**Fig. 3:**
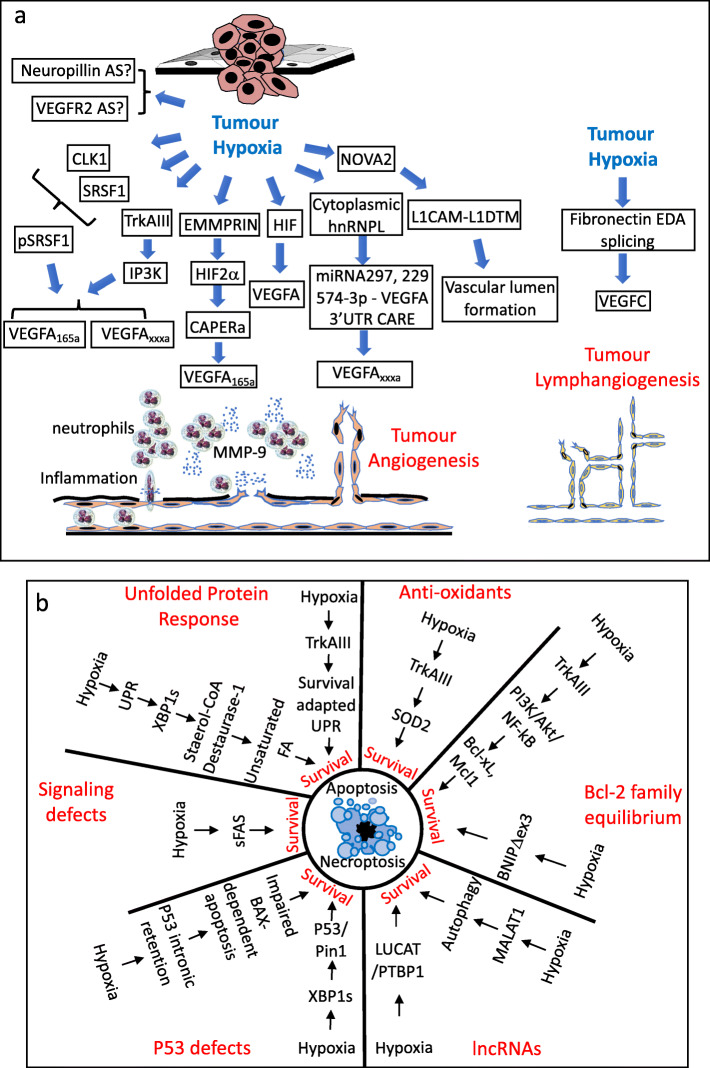
Tumour hypoxia-induced alternative splicing and angiogenesis and surviving programmed cell death. Schematic representations of the role of tumour hypoxia-induced alternative splicing (AS) in: **a** tumour angiogenesis and **b** tumour cell evasion of programmed cell apoptotic and necroptotic cell death

Hypoxia-induced alternative splicing drives angiogenesis and is, therefore, fundamental for tumourigenicity, clonal expansion and metastatic progression [51, 131]. Under normoxic conditions, HIF-α subunits are proline hydroxylated by PHD prolyl-hydroxylase, complex with pVHL, elongin B, elongin C, Cullin2 and Rbx1 and are directed for proteasomal degradation. Hypoxia inactivation of PHD results is dissociation of HIFα/pVHL complexes, resulting in HIF-1α and HIF-2α accumulation and stabilization, nuclear translocation, heterodimerization with nuclear ARNT subunits to form HIF-1α/ARNT (HIF-1) and HIF-2α/ARNT (HIF2) transcription factors and the induction of HIF-dependent pro-angiogenic alternative VEGFA and VEGFR receptor expression and splicing [33, 36–40, 44, 132, 133]. In this mechanism, hypoxia reduces the ratio of fully spliced long form HIF-1αL that exhibits weak transcriptional activity, to alternatively spliced short form HIF-1αs, augmenting pro-angiogenic HIF-1-dependent VEGFA and VEGFR2 transcription, angiogenesis and alternative splicing of HIF-target and non-target genes [36, 134, 135]. Furthermore, hypoxia induces pro-angiogenic *VEGF-A* gene alternative splicing, resulting in VEGF-A_111_, VEGF-A_121_, VEGF-A_145_, VEGF-A_165_, VEGF-A_183_, VEGF-A_189_ and VEGF-A_206_ isoform expression. VEGF-A_145_, VEGF-A_189_ and VEGF-A_206_ bind strongly to cell surfaces and matrices, VEGF-A_111_ and VEGF-A_121_ lack exons 6 and 7 and are diffusible, whereas VEGF-A_165_ is partially diffusible, matrix-associated and is a more potent angiogenesis inducer due to its heparin binding capacity that facilitates interaction with angiogenic neuropilin VEGFR co-receptors. Alternative *VEGFA* splice variants derived from exon 8 alternative splicing also include angiogenesis promoting VEGFA_xxxa_ and inhibiting VEGFA_xxxb_ isoforms, both of which bind VEGFR2 but only the VEGFA_xxxa_ isoform activates angiogenic signaling. Alternative VEGFA_xxxa_ and VEGFA_xxxb_ splicing depends upon SRSF1 and SRSF6 splice factors, as SRSF6 selects the exon 8a distal 5′ splice site resulting in VEGFA_xxxb_ expression and SRSF1 selects the exon 8a proximal splice site resulting in VEGFA_xxxa_ expression. The hypoxia regulated splicing factor kinase SRPK1 phosphorylates SRSF1 to promote exon 8a inclusion and VEGFA_xxxa_ expression, and either SRSF1 or SRPK1 repression promote VEGFA_xxxb_ expression. Hypoxia also promotes the expression and activation of SRSF1, SRPK1 and CLK1 splicing factor kinases, providing indirect hypoxia-inducible alternative VEGFA_xxxa_ splice mechanisms for promoting angiogenesis (Fig. 3a) [51, 136].

HIF-1-target genes involved in angiogenesis also include the extracellular matrix metalloproteinase inducer EMMPRIN, which is up-regulated by hypoxia in cancer cells [137]. EMMPRIN promotes HIF-2α expression and activates the AP1, ERα and ERβ transcriptional co-regulator CAPER-α, shifting VEGF-A_189_ to VEGF-A_165_ expression [138, 139]. Hypoxia also promotes cytoplasmic translocation of ribonucleoprotein L, which competes with miR-297, miR-299 and miR-574-3p microRNAs to target the CA rich (CARE) element in the VEGFA mRNA 3′-UTR, providing an additional mechanism through which hypoxia can promote VEGFA isoform expression by inhibiting miRNA binding [140]. In addition, hypoxia also reduces MAX transcription factor expression through alternative intron-inclusion splicing, altering angiogenesis dependent upon MAX interaction with lncRNA EGFL7OS, involved in pro-angiogenic VEGF-A_165_ alternative splicing (Fig. 3a) [141].

In human neuroblastoma cells, the hypoxia-inducible oncogenic alternative TrkAIII splice variant also promotes angiogenic alternative VEGF-A_165_ splicing via PI3K-signaling, increasing tumour xenograft growth and vascularization in vivo [89]. In muscle cells, hypoxia-induced VEGF expression also depends upon the alternatively spliced peroxisome proliferator-activator receptor γ coactivator alpha isoform NT-PGC-1α, required for endothelial cell migration and tube formation, with implications for angiogenesis in myosarcomas [142, 143].

VEGF receptors (VEGFRs) also exhibit hypoxia-induced alternative splicing. In endothelial cells, hypoxia increases the ratio of VEGFR-1 non-signaling decoy VEGFA receptor and expression of a truncated soluble sVEGFR-1 alternative intron-retained splice variant, comprised of the first 13–14 exons, that complexes with VEGFR2 to reduce VEGF signaling [144]. VEGFR-2, which binds all VEGFA isoforms, is the major signaling receptor involved in angiogenesis and is also expressed as an alternative soluble sVEGFR-2 splice isoform that inhibits VEGFC/VEGFR3-dependent lymphoangiogenesis. Although a direct role for hypoxia in sVEGFR-2 and soluble s-neuropilin VEGF co-receptor [145] alternative splicing has not been reported, the sVEGFR-2 isoform is induced by inflammatory cytokines IL-8 and IL-12 in human benign prostate hypertrophy tissue microvascular endothelial cells, suggesting an indirect role for hypoxia-induced inflammation in down-regulating angiogenesis in benign prostate tumours through alternative sVEGFR2 splicing [146]. Hypoxia also increases endothelial cell expression of the splice factor NOVA2, which is overexpressed in colon and ovarian cancers, and regulates endothelial cell polarity, vascular lumen formation, and also promotes expression of a soluble L1-ΔTM L1CAM alternative splice isoform, which stimulates angiogenesis and promotes ovarian cancer progression [147, 148]. The VHL HIF1α inactivator and tumour suppressor also exhibits hypoxia-regulated alternative splicing, characterized by inactivating mutations in the cryptic exon (E1) deep in intron 1 that promotes excessive E1 retention and VHL protein repression, within the context of pre-neoplastic von Hippel Landau disease, leading to aberrant HIF activation (Fig. 3a) [149].

Hypoxia induction of the UPR, resulting alternative unconventional XBP1s splicing, promotes XBP1s complexing with HIF-1α, influencing HIF-1 transcriptional function by recruiting RNA polymerase to the promoters of pro-angiogenic VEGFA, metabolic PDK1 and GLUT1 regulators, and DDIT4 negative mTOR regulators, increasing their expression in triple-negative breast cancer (TNBC) [150]. Furthermore, hypoxia also induces an alternative intron 3-skipped splicing event in the *Cysteine rich 61 (Cyr61*) gene, resulting in a secreted, biologically active, pro-angiogenic Cry61 isoform that promotes breast carcinogenesis [151]. Finally, in a recent exon array analysis, 9 novel hypoxia-induced alternative splice events have been detected in the endothelial cell angiogenesis-associated cytoskeleton remodeling genes *cask, itsn1, larp6, sptan1, tpm1* and *robo1* [152].

Hypoxia-induced alternative splicing has also been implicated in tumor-induced lymphangiogenesis in mouse xenografts, inducing the expression of the alternative extra domain A fibronectin (EDA-FN) isoform that increases lymphangiogenesis by promoting VEGFC expression, and also promotes stem cell proliferation [153, 154], implicating hypoxia-induced alternative EDA-FN splicing in lymphatic metastatic dissemination and cancer stemness (Fig. 3a).

## Hypoxia-induced alternative splicing in survival, and evasion of programmed cell death (hallmark 5)

The induction of programmed cell death is a fundamental tumour suppressing mechanism that results from two well characterized caspase-dependent apoptotic pathways, the intrinsic mitochondrial pathway and the extrinsic cell surface pathway, both of which involve the effector caspases 3 and caspase 7 [155]. Tumour cell survival and tumour progression, therefore, involves evasion of programmed cell death mechanisms also influenced by hypoxia-induced alternative splicing [156].

In brief, the intrinsic mitochondrial apoptosis pathway is activated by pro-apoptotic members of the Bcl-2 family that permeabilize the outer mitochondrial membrane, resulting in the release of mitochondrial pro-apoptotic cytochrome c, Smac/Diablo and HTR2A proteins into the cytoplasm, which inactivate cytoplasmic apoptosis inhibitory cAIP1 and cAIP2 proteins, inducing cleavage-activation of pro-apoptotic caspases, 3, 7 and 9. In contrast, the extrinsic apoptosis pathway, activated principally by NK and cytotoxic T lymphocyte populations, involves death-inducing TNF-family ligands TNFα, FASL and TRAIL, which bind TNFR, FAS and DR4/5 TRIAL death receptors, induced on the surface of damaged cells by activated tumour suppressors, such as TP53. Death receptor ligation promotes death-inducing signaling complex formation, resulting in cleavage-activation of caspases 8 or 10, leading either to direct cell-death via effector caspases 3 and 7 or, in conditions of low caspase 8 activity, indirect activation of the intrinsic mitochondrial apoptosis pathway via caspase-8-dependent tBid cleavage and mitochondrial translocation, resulting in tBid/Bax-dependent outer-mitochondrial membrane permeabilization and apoptosis via the mitochondrial pathway [156, 157].

With respect to the impact of hypoxia-induced alternative splicing on apoptosis [158], in breast cancer cells, chronic hypoxia promotes alternative intron 1-retention splicing in the TNF family member *TNFSF13*, resulting in suppression of TNFSF13 anti-apoptotic activity, implicating hypoxia-induced TNFSF13 alternative splicing in tumour suppression [56]. In hepatocellular carcinoma cells, hypoxia induces alternative exon 6 skipped FAS splicing, resulting in a soluble isoform deleted of the transmembrane domain, that inhibits Fas-dependent apoptosis [159]. Hypoxia also increases the ratio of fully spliced anti-apoptotic long Bcl-xL to alternatively sliced apoptosis-promoting short form Bcl-xS in cancer cells [160–163], and up-regulates alternative exon 3-skipped *BNIP3* splicing, resulting in BNIP3 ΔEx3 isoform expression that is devoid of a mitochondrial localization signal and competes with pro-apoptotic fully spliced BNIP3 to promote survival [164–166]. In neuroblastoma cells, the hypoxia-regulated alternative TrkAIII splice variant induces survival PI3K/Akt/NF-κB signaling, increases anti-apoptotic Bcl-xL and Mcl-1 expression, enhances resistance to oxidative-stress by augmenting mitochondrial SOD-2 expression and activity and increases survival under conditions of acute ER-stress by activating a modified survival-adapted UPR [89, 90, 167–169]. In breast cancer cells, hypoxia-sensitive hnRNPs also induce alternative Mcl1 splicing [170], lncRNA LUCAT-1 expression, LUCAT-1 complexing with PTPB1 splice factor promoting survival and therapeutic-resistance [100] and alternative intron retention splicing and NMD inactivation of TP53, resulting in evasion of TP53 and BAX-dependent apoptosis [56]. In Myc-dependent cancers, UPR activation and unconventional alternative XBP1s splicing, results in XBP1s target gene transcription, increasing the expression of stearoyl-CoA-desaturase 1 and unsaturated fatty acid levels, promoting survival [113]. In osteosarcoma cells, XBP1s splicing promotes PI3K/mTOR survival signaling and in glioma cell is essential for maintaining hexokinase II expression, ATP production, anti-apoptotic Bcl2 expression and inhibitory TP53/Pin1 complexing [115]. Consistent with a role for HIFs in cancer cell survival, UPR activation also promotes cooperation between XBP1s and HIF-1α increasing survival [171]. UPR-dependent PERK activation also promotes survival by reducing protein synthesis via inhibitory e1F2α phosphorylation [172], which involves alternative *E1F2B5* intron-retention splicing and expression of dominant negative E1F2B5ε, which substitutes E1F2B5 in E1F2B complexes, reducing e1F2α-dependent translational initiation. E1F2B5ε is overexpressed in head and neck cancers, implicating hypoxia-induced E1F2B5ε splicing in reducing protein expression and promoting survival during periods of hypoxia-induced acute and chronic ER stress [35]. Hypoxia also suppresses the expression exon 3 and 4 skipped Mushash-1 RNA binding protein in cancer cells, enhancing survival and resistance to cisplatin cytotoxicity [173], and the atypical splicing factor SRSF10 also plays a central role in promoting the expression of alternatively spliced stress- and apoptosis-associated genes, promoting survival under ER-stress conditions (Fig. 3b) [174].

In addition to apoptosis, programmed necroptotic tumour cell-death is also influenced by hypoxia-induced alternative splicing. This caspase-independent cell death mechanism is characterized by cellular vacuolation, cellular swelling and necrotic cell lysis is mediated by RIPK1, RIPK3 and MLKL, induced by the UPR, and also involves unconventional XPB1s splicing and hypoxia-induced autophagy, which regulate autophagosome/lysosome fusion [5, 175]. Within this context, hypoxia induced expression of the master splice-regulator lncRNA MALAT1 promotes a pro-survival autophagic response [176], associated with hypoxia repressed SRSF3 splicing factor expression, implicating yet to be defined splicing alterations in the inhibition of BECN1 autophagy suppressor expression (Fig. 3b) [177].

## Hypoxia-induced alternative splicing in immune evasion (hallmark 6)

Tumour progression also depends largely upon evasion of anti-tumor immunity, and hypoxia-induced alternative splicing plays a critical role in de-regulating the anti-tumour immune response [178].

**Fig. 4:**
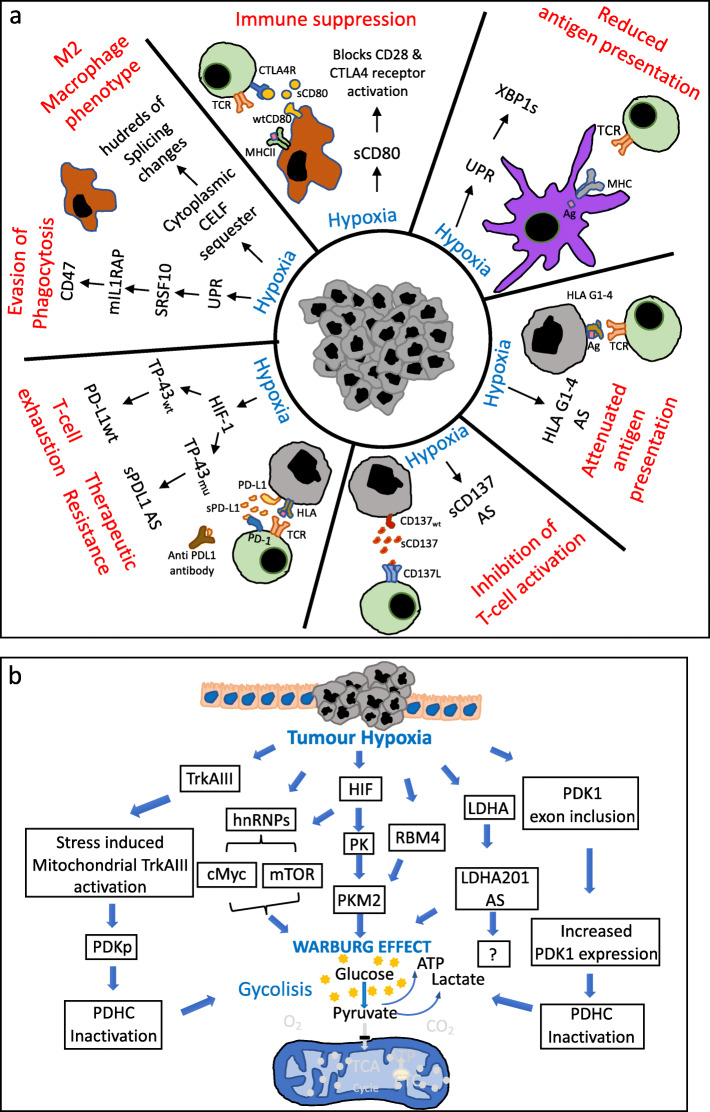
Tumour hypoxia-induced alternative splicing (AS), inflammation, immunity and metabolic adaptation. Schematic representations of tumour hypoxia-induced alternative splicing mechanisms that: **a** protect tumour cells from anti-tumour immunity and inflammation and; **b** that impacts tumour metabolism to promote the glycolytic “Warburg Effect”

Hypoxia induces alternative splicing of the co-stimulatory TNFR family member *CD137,* reported in a variety of tumour cell types, results in the expression of soluble sCD137 that binds CD137L, inhibiting interaction with wtCD137 and preventing T-lymphocyte activation [179]. Hypoxia promotes alternative splicing of *HLA-G* human leukocyte antigen G, a non-classical major histocompatibility complex (MHC) class I immune checkpoint molecule, resulting in expression of 4 membrane bound (HLA-G1-G4) and 3 soluble (HLA-G5-G7) isoforms in melanoma, choriocarcinoma, lymphoma, glioma and other cancer cell types, that attenuate NK, cytotoxic T-cell and antigen presenting cell activity [180]. UPR-dependent unconventional alternative XBP1s splicing drives dendritic cell (DC) malfunction, is maintained within tumour microenvironments, disrupts DC homeostasis, alters local antigen-presenting capacity, promotes evasion from T-cell mediated protective anti-tumour immunity and facilitates tumour progression [181]. The hypoxia-regulated PDL1 suppressor of adaptive immunity is also expressed as 2 soluble alternative splice variants in human non-small cell lung carcinoma, in association with mutation of TDP-43 splicing factor, which regulates PD-L1 expression and splicing. Both soluble PD-L1 isoforms bind PD-1, act as PD-1 decoys, promote lymphocyte exhaustion and enhance resistance to anti-PD-L1 immune-therapy (Fig. 4a) [182, 183].

## Hypoxia-induced alternative splicing in metabolic reprogramming (hallmark 7)

Hypoxia modifies metabolism and is a critical component in maintaining the glycolytic metabo-type that characterizes malignant tumour progression [184].

Otto Warburg was the first to observe that malignant tumours rely upon glycolysis for their metabolic and anabolic needs and process glucose to pyruvate and lactate via glycolysis [1]. Hypoxia-induced alternative pre-mRNA splicing influence on tumour metabolic reprogramming takes its initial cues from physiological metabolic reprogramming under anaerobic conditions, which initiates with HIF1α stabilization and HIF1 promotion of glycolysis-promoting alternative pyruvate kinase PKM2 splice variant isoform expression, at the expense of the oxidative phosphorylation-promoting PKM1 isoform, resulting in a metabolic shift to glycolysis. Although malignant tumours eventually acquire a continuous glycolytic metabolic state under anaerobic conditions, during tumourigenesis and throughout tumour progression, hypoxia remains a fundamental condition that promotes glycolysis in both normal and neoplastic tumour components.

Pyruvate kinase (PK) catalyzes phosphate transfer from phosphoenolpyruvate to ADP, producing 1 molecule of pyruvate and 1 molecule of ATP, providing carbons for the mitochondrial citric acid cycle. The *PK* gene exhibits alternative splicing and is expressed as liver PKL, erythrocyte PKR, adult tissue PKM1 and lung, adult stem cell, embryonic and tumour PKM2 isoforms. PKM1 and PKM2 represents alternative splice variants of the same 12 exon transcript, in which exons 9 and 10 are mutually exclusive. Hypoxia promotes HIF-1-dependent PK expression and alternative PKM2 splicing [185, 186]. PMK2 exhibits weaker enzymatic activity than PMK1, resulting in accumulation of glycolytic intermediates for biomolecular synthesis, providing an initial hypoxia-induced alternative splice input for metabolic reprograming during tumour initiation, early expansion and progression. This evolves into more-permanent HIF-dependent alternative PKM2 splicing under aerobic conditions, as oncogenes are activated that promote constitutive hypoxia-independent HIF expression and activity, alter hnRNPs or c-Myc expression or induce mTOR signaling, resulting in the constitutive PKM2 expression that characterizes the “Warburg effect” in a wide range of malignancies [1, 187]. The hypoxia-regulated RBM4 splicing factor also promotes alternative PKM2 splicing in embryonic stem cells, with potential implications for cancer stem cell metabolism (Fig. 4b) [188].

Pyruvate, the end product of glycolysis, is a major substrate for oxidative metabolism and a branching point for glucose, lactate, fatty acid and amino acid synthesis [189, 190]. During oxidative metabolism, cytosolic pyruvate is transported to the inner mitochondrial membrane by MPC1 and MPC2 pyruvate carriers, where it is oxidized by the pyruvate dehydrogenase complex (PDHC) to Acetyl CoA prior to entering the TCA cycle, in which carbons are converted to CO2 and energy (NADH, NADPH and ATP) [187, 191]. Mitochondrial PDHC links and controls the flux of pyruvate from glycolysis to the TCA cycle and catalyzes irreversible pyruvate conversion to acetyl-CoA. Hypoxia inactivates PDHC, providing pyruvate for lactate dehydrogenase A (LDHA), which catalyzes reversible conversion to lactic acid [192]. LDHA is a HIF-target gene and key to the “Warburg effect”, producing lactate and NAD+ for both aerobic and anaerobic glycolysis, this enzyme is up-regulated by hypoxia and exhibits hypoxia-induced alternative splicing. In breast cancer cells, acute and chronic hypoxia promote alternative LDHA-001 (alternative first exon) splicing and reduce LDHA-201 (intron 1-retained) isoform expression, leading to loss of LDHA-201 expression through NMD. However, the influence of this on metabolism remains to be elucidated (Fig. 4b )[56].

The hypoxia-regulated alternative TrkAIII splice variant also promotes stress-induced metabolic reprogramming in human neuroblastoma cells, by localizing to mitochondria under non-stressed conditions in inactive form, where it exhibits mitochondrial internalization and cleavage-dependent activation under conditions of ER stress, resulting in tyrosine phosphorylation of pyruvate dehydrogenase kinase (PDK1) and glycolytic metabolic re-programming [193].

Hypoxia-induced PDK2 activation also associates with hypoxia-induced alternative BNIP3Δex3 splicing, linking metabolic re-programming to survival [164, 165, 194], and the hypoxia-regulated SR protein SC35 induces aberrant E1a pyruvate dehydrogenase splicing promoting acidosis within the hypoxic microenvironment [195]. In hepatocellular carcinoma cells, HIF-dependent alternative exon inclusion splicing of pyruvate dehydrogenase kinase PDK1 is also induced by hypoxia, promoting glycolysis via this important pyruvate dehydrogenase complex inhibitor (Fig. 4b) [36].

## Hypoxia-induced alternative splicing in EMT, tumour invasion, metastasis and stemness (hallmark 8)

Tumour invasion and metastasis is a multistep process in the majority of carcinomas and is characterized by tumour cell breaching of basement membrane barriers, motility, invasion of local tissues, systemic dissemination via lymphatic and blood vessels, arrest in microvasculature of distant organs and metastatic growth. This process is facilitated by the accumulation of genetic mutations and is promoted by hypoxia and hypoxia-induced alternative splicing.

**Fig. 5:**
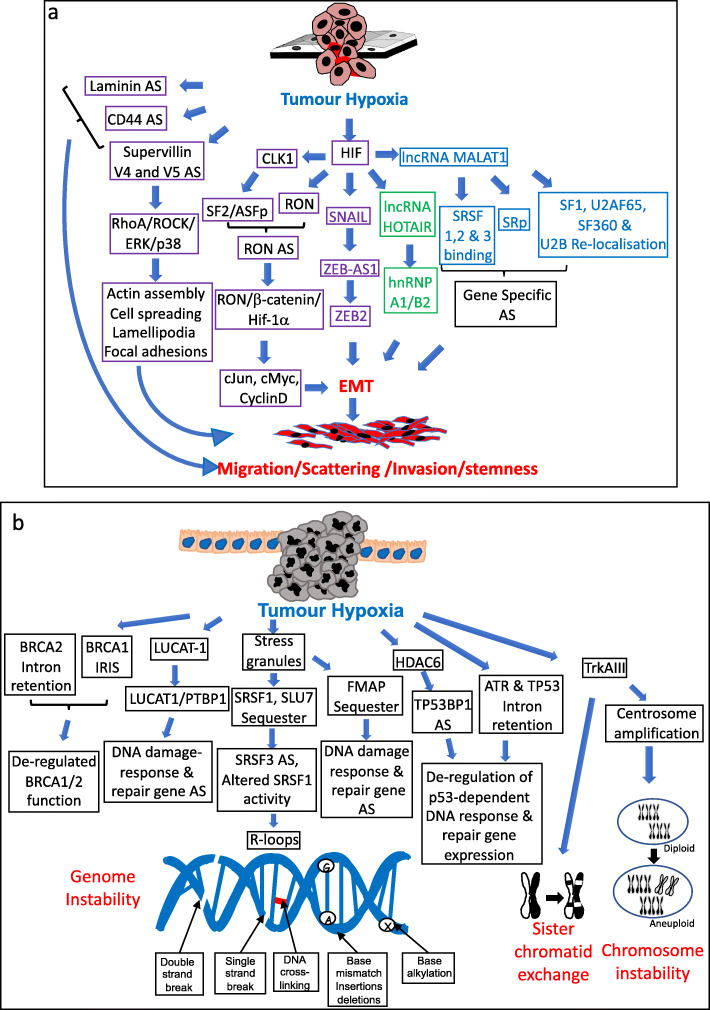
Tumour hypoxia-induced alternative splicing, EMT, invasion and genomic instability. Schematic representations of the many ways that tumour hypoxia-induced alternative splicing (AS) promotes: **a** epithelial to mesenchymal transition (EMT), tumour cell migration, scattering and invasion during tumour progression; and **b** genomic and chromosomal instability

In the majority of carcinomas, this multi-step process initiates with an adaptive metaplastic transition from an epithelial to mesenchymal phenotype (EMT), characterized by hypoxia-triggered loss of epithelial cell polarity and cell-cell adhesive interactions, acquisition of migratory invasive behavior and a gene expression profile more characteristic of multipotent mesenchymal stromal cells [196]. Several hypoxia-induced alternative splicing events have been closely linked to EMT. In hepato-carcinoma cells, hypoxia induces alternative splicing of the membrane and actin-associated protein Supervillin, involved in actin filament assembly, cell spreading, lamellipodia extension and regulation of focal adhesions, resulting in V4 and V5 alternatively spliced isoforms that promote RhoA/ROCK-ERK/p38-dependent EMT [197]. In a variety of cancer cell types, hypoxia induces expression of lncRNAs MALAT1, ZEB2-AS1 and HOTAIR, which are master regulators of alternative splicing, miRNA sponging, EMT, invasion, migration, cancer staminality and metastatic growth [66, 68–70, 198–203]. MALAT-1 localizes to nuclear speckle pre-mRNA splicing sites, interacts with SRSF1, SRSF2, and SRSF3 splice factors, influences SF1, U2AF65, SF3a60, and U2B distribution and modulates SR splice factor phosphorylation [204, 205], resulting in gene-specific EMT-promoting alternative splicing and metastatic progression in colorectal and triple negative breast cancers [67, 206]. Hypoxia-induced HOTAIR expression interacts with the B1 component of the heterogeneous ribonuclear matchmaker protein HnRNP A2/B1 and regulates RNA/snRNA annealing to specific pre-mRNA splicing targets, altering splicing and promoting EMT, tumour invasion and metastasis [207]. Hypoxia also induces the EMT-promoting transcription factor Snail [208, 209], which stimulates ZEB2-AS1 lncRNA expression, preventing ZEB2 mRNA intron 1 alternative splicing, a critical event in ZEB2 protein expression [199] and ZEB2-dependent repression of E-Cadherin expression, EMT and proliferation, and also impairs apoptosis by repressing Bax, caspase 3 and caspase 9 expression (Fig. 5a) [210].

The HIF-1-target proto-oncogene RON has also been implicated in hypoxia-induced EMT, tumor invasion and metastasis. Hypoxia induced oncogenic alternative RON splicing and activation promotes CLK1-medited SF2/ASF splice factor phosphorylation-dependent reduction in E-cadherin expression, and promotes actin reorganization and vimentin expression, resulting in EMT, invasion and metastasis. Hypoxia induced oncogenic alternative spliced RON isoforms associate with breast, lung, liver, kidney, bladder, ovarian, colon, pancreatic, gastric and prostate carcinoma progression and, in contrast to dominant negative RON isoforms, in general, promote EMT, tumour cell migration, scattering, invasion and metastatic progression (Fig. 5a )[99].

Hypoxia-induced EMT also associates with repression of ESRP1 splice factor expression, resulting in α6B integrin subunit alternative splicing and the generation of α6Bβ1 integrin receptors that characterize cancer stem cell phenotypes [211, 212]. At present, however, it is unclear whether hypoxia is also responsible for expression of the novel E-Cadherin splice variant Ecadvar, detected in several cancer cell lines, which down regulates wild type E-cadherin expression in breast cancer cells, decreasing cell-cell interactions, increasing motility and enhancing invasive capacity [213].

EMT also associates with a progressively more cancer stem cell-like phenotype [214–216], reported to involve interactions between HIF-1α, ZEB1 and the soluble sCD44 splice variant, implicating hypoxia-regulated alternative splicing in cancer stem cell promotion [217, 218]. Within this context, severe hypoxia exerts cell-specific effects upon gene expression and alternative splicing [219], including the expression of DCLK1 splice variants that promote stem cell self-renewal and drug-resistance [220], and the hypoxia-induced alternative TrkAIII splice variant that promotes and maintains a cancer stem cell-like phenotype in human neuroblastoma cells (Fig. 5a) [168, 221].

In head and neck cancers, hypoxia-induced laminin α3 chain alternative splicing and expression of the splice variant LAMA3 isoform has been implicated in tumour invasion and metastatic progression [222] as has hypoxia alteration of the PTBP-1-regulated alternative splice equilibrium between invasion and motility promoting protein cortactin and its invasion/migration inhibiting alternative exon 11 inclusion spliced isoform (Fig. 5a )[223, 224].

## Hypoxia-induced alternative splicing in tumour-associated inflammation (hallmark 9)

Tumour initiation, rapid expansion and microenvironmental hypoxia, are accompanied by an acute inflammatory response that is regulated by complex NF-κB signaling [225]. Tumours recruit inflammatory leucocyte and lymphocyte populations that are essential for tumour angiogenesis, which are manipulated and subverted within the tumour microenvironment to promote rather than impede tumor progression. In this process, tumor chemical and cellular micro-environments interact to promote tumor promoting N1 neutrophil and M2 macrophage phenotypes, which can be reverted to tumour inhibiting N2 and M1 phenotypes by relieving tumour hypoxia [226, 227]. Tumour associated macrophages (TAMs) make up significant proportions of most tumours, accumulate within hypoxic/necrotic areas in endometrial, breast, prostate and ovarian carcinomas and promote aggressive tumor behavior and metastatic progression [228]. Hypoxia suppresses the M1 macrophage anti-tumor pro-inflammatory phenotype [229–233] by promoting cytoplasmic stress granule sequestration of splicing factors, including CELF1, helping to explain why M1 macrophages express hundreds of spliced RNAs not expressed by M2 tumour-promoting TAMs, implicating hypoxia-induced cytoplasmic CLEF-1 retention in promoting the alternative splicing events that promote and maintain the M2 macrophage tumour promoting phenotype [234]. Hypoxia also suppresses adaptive immunity by reducing cell surface expression of the monocyte/macrophage co-stimulatory molecule CD80 and promoting alternative CD80 splicing, resulting in expression of a soluble sCD80 isoform that binds and blocks CD28 and CTLA4 receptor activation, resulting in immune suppression [235]. UPR-induced unconventional XBP1s splicing increases hepatic pro-inflammatory cytokine IL-6 expression and secretion, promoting autocrine/paracrine STAT3 activation-dependent hepatocellular carcinoma growth [116], and has been implicated in antagonizing NF-kB-dependent pro-inflammatory cytokine expression and secretion to repress acute inflammation in some cancers, reducing anti-tumoral activity [236]. In cervical tumour cells, the UPR also induces oncogenic activation of the atypical splicing factor SRSF10, resulting in *IL1-RAP* alternative exon 13 inclusion, membrane associated mIL1-RAP expression and IL1β/IL1R1/mIL1RAP-dependent expression of CD47, the “don’t eat me” inhibitor of macrophage phagocytosis, identifying a UPR/SRSF10/mIL1RAP/CD47-dependent tumour-promoting axis (Fig. 4a )[237].

## Hypoxia-induced alternative splicing in tumour genetic instability (hallmark 10)

Genetic instability underpins all stages of cancer, from tumour initiation to metastatic disease, and is both directly and indirectly influenced by tumour hypoxia-induced alternative splicing.

Hypoxia induces stress-dependent re-localization of RNA binding proteins, spliceosome components and splicing factors to stress granules in an indirect mechanism that promotes R-loop formation, as a co-lateral active transcriptional consequence of nascent RNA hybridization to the DNA template. R-loops destabilize the genome, halt DNA replication, promote double strand DNA breaks and are prevented by RNaseH1, RNA-DNA helicases, topoisomerases, mRNA ribonucleoprotein (mRNP) biogenesis factors and by SRSF1 and Slu7 splicing factors. Hypoxia promotes SRSF1 and Slu7 cytoplasmic stress granules sequestration, reducing nuclear levels and resulting in mitotic aberrations, R-loop formation and genomic instability, characterized by DNA-damage, mitotic derangement and sister chromatid cohesion, dependent upon aberrant SRSF1 (ASF/SF2) splicing factor activity, alternative SRSF3 truncated SRp20 and -TR isoforms expression [238–240]. Hypoxia also promotes cytoplasmic stress granule sequestration of the spliceosome component MFAP1 [241], reducing nuclear MFAP1 levels, resulting in alternative splicing of DNA damage response and DNA repair genes that results in genomic instability [242]. Additional mechanisms by which hypoxia-regulated alternative splicing promotes genetic instability, include induction of LUCAT1 expression and complexing with PTBP1, resulting in inhibitory alternative DNA damage-related gene splicing, and inhibitory intron-retention alternative splicing of DNA damage and DNA repair pathway genes in human colorectal and breast cancer cells [56]. Hypoxia also switches DNA damage response pathway coding transcripts to non-coding intron-retained alternative spliced transcripts in genes, such as HDAC6, a cytotoxic response regulator that regulates inhibitory alternative splicing of the TP53BP1 p53 binding protein and TP53 co-factor, resulting in de-regulated double strand DNA repair in colorectal cancers, highlighting a predominant role for hypoxia-induced alternative splicing in de-regulating the DNA damage and DNA repair responses (Fig. 5b )[118].

In breast cancer cells, hypoxia also triggers alternative BRCA1-IRIS splicing in hypoxic/necrotic niches, promoting tumour progression by de-regulating wtBRCA1 function [243–247], and also inactivates TP53, ATR, BRCA2 and Bax tumour suppressors by promoting alternative intronic retention splicing and NMD, reducing TP53, ATR and BRCA2 involvement in the DNA damage response [56].

Finally, expression of the hypoxia-regulated alternative TrkAIII splice variant in neuroblastoma cells augments sister chromatid exchanges and re-localizes to centrosomes in active form, inducing polo kinase 4 activation, centrosome amplification, enhanced tubulin polymerization and chromosomal instability (Fig. 5b )[221, 248].

## Therapeutic prospects

Between 10 and 30% of solid tumours are characterized by fluctuating acute and chronic hypoxia, resulting in cellular hypoxic responses that include alternative pre-mRNA splicing and the expression of novel protein isoforms that promote tumour progression and impact therapeutic efficacy. Hypoxic regions of tumours are populated by slowly dividing tumour cells that escape death induced by cytotoxic agents that target proliferating cells and are infiltrated by immature tortuous permeable blood and lymphatic vasculatures that increase tumour interstitial hypertension, a potent force for drug expulsion. Tumour glycolytic adaptation renders the hypoxic tumour microenvironment acidic and reducing, further de-regulating inflammatory and immune cell recruitment and function, enhancing multidrug resistance through elevated expression of p-glycoprotein multidrug transporter, which combined with mechanisms to evade programmed cell death, greatly reduce therapeutic efficacy. Considering the many roles of hypoxia-induced alternative splicing in tumour pathogenesis and progression, targeting tumour microenvironmental hypoxia, the tumour microvasculature, hypoxic responses, hypoxia-induced alternative splicing and tumour promoting alternative splice protein isoforms, are all of potential therapeutic importance [249–257].

### Targeting tumour hypoxia - tumour reoxygenation and vascular normalization

Therapeutic efficacy can be enhanced by interfering with or reprogramming the hypoxic tumour niche to improve drug efficacy [258]. Tumour reoxygenation improves fractionated radiotherapeutic efficacy and can be achieved by hyperbaric oxygenation, intra-tumoral injection of lipid stabilized oxygen microbubbles that enhance tumour oxygenation and radiotherapeutic efficacy in rodent tumour models [259, 260], by nanoparticle-mediated tumor reoxygenation and oxygen-generating methods [261] or by artificial red cells [8].

**Fig. 6:**
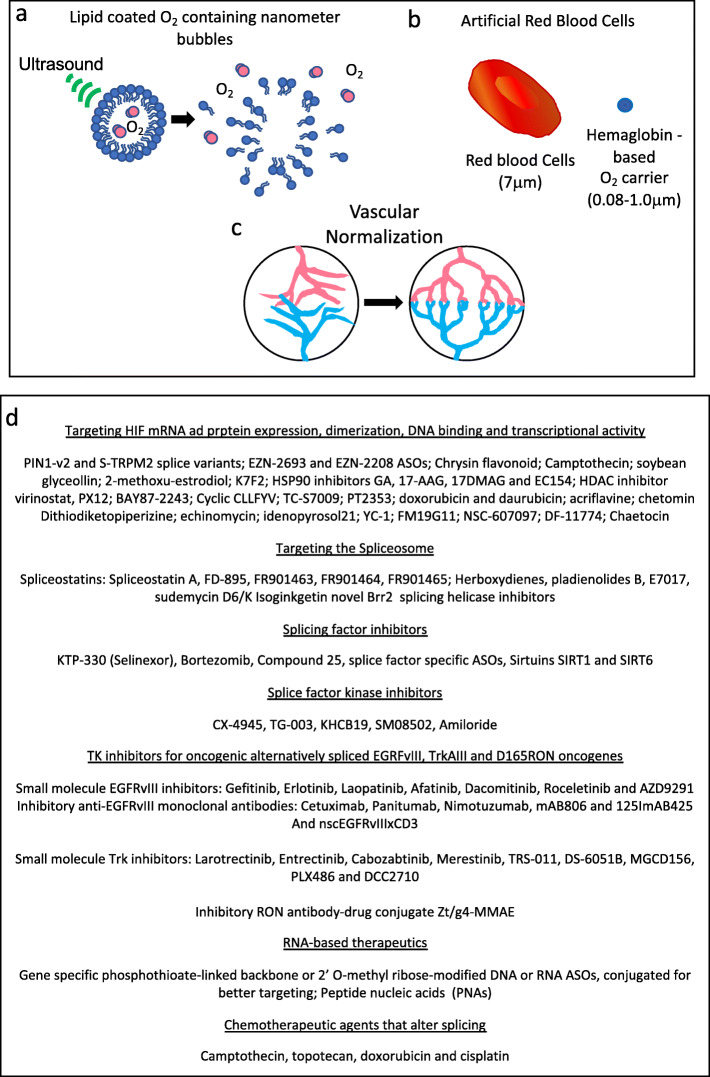
Tumour hypoxia-induced alternative splicing: Therapeutic approaches. Schematic representation of the potential therapeutic approaches for reducing tumour hypoxia and subsequent tumour promoting alternative splicing, including **a** The use of lipid coated oxygen containing microbubble or nanoparticles that can be induced to release oxygen within hypoxic tissue by ultrasound (and also by diffusion, not shown); **b** Artificial red blood cell hemoglobin based oxygen carrying particles of 0.8–1.0 μm which reach places that 7 μM red blood cells cannot; **c** Vascular normalization by subtle re-equilibration of angiogenic equilibria, and **d** Small molecule inhibitors and modified antisense oligonucleotides and peptide nucleic acids to target spliceosome components, splicing factors, splice factor kinases, hypoxia induced alternatively spliced tyrosine kinase oncogenes and chemotherapeutic agents that alter splicing

“Normalization” of the aberrant tumour vasculature is also emerging as an alternative way to improve tumour oxygenation, reduce tumour progression and therapeutic efficacy. This stems from observations that vascular destruction by anti-angiogenic agents promotes tumour hypoxia, reduces therapeutic efficacy and facilitates metastatic progression. The tumour microvasculature is immature, permeable, tortuous, haphazard, exhibits aberrant basement membranes and lacks a complete repertoire of cellular and matrix components required for vascular maturation and function. This flawed system increases interstitial hypertension resulting in drug expulsion, inducing selection of more aggressive phenotypes through adaptation to hypoxia, which is facilitated by hypoxia-induced alternative splicing. Vascular “Normalization” requires the delicate rebalancing of angiogenic factor/inhibitor equilibria and can be achieved by careful selection and dosage of antiangiogenic agents. This has been demonstrated by down regulating VEGFA expression in a human tumour mouse xenograft model, resulting in the pruning immature permeable vessels, re-modeling a less-permeable, less-tortuous vasculature with more pericytes and near-normal basement membrane, responsible for increasing tumour oxygenation, decreasing tumour interstitial pressure and improving drug penetration [243]. Consistent with this, patients treated with the monoclonal VEGFA inhibitor bevacizumab or with small molecule PTK787 and SU6668 VEGFR tyrosine kinase inhibitors, exhibit improved tumour blood flow, reduced tumour microvascular density, volume and tumour interstitial pressure but do not exhibit decreased radioactive tracer uptake, indicating improved drug-uptake potential. This effect, however, may be short lived and requires better understanding of the molecular mechanisms involved in order to prolong this effect [245]. PHD2 inhibition also promotes tumour vascular “normalization”, restoring tumor oxygenation, normalizing the vascular endothelium and inhibiting metastatic progression (Fig. 6a )[262].

### Targeting hypoxia-induced alternative splicing

Tumour promoting subversion of pre-mRNA splicing by hypoxia, resulting in oncogene activation, tumour suppressor inactivation, immortalization, metabolic adaptation, evasion of programmed cell death and anti-tumour immunity, angiogenesis, tumour-promoting inflammation and genetic instability, not only depends upon specific alternatively spliced protein isoforms but also specific spliceosome components, splicing factors, splice factor kinases and splicing, all of which represent potential therapeutic targets.

#### HIF inhibitors

HIF transcription factors are activated by tumour hypoxia, promote HIF-target expression and alternative splicing of HIF-target and non-HIF target genes [36]. Hypoxia inactivation of PHD proline hydroxylase, results in dissociation of HIFα/VHL-VEC complexes, HIFα stabilization, nuclear translocation and hetero-dimerization with ARNT/HIFβ components [37, 38], identifying many relevant therapeutic targets.

HIF inhibitors fall into different categories and include inhibitors of HIF mRNA and protein expression, inhibitors of HIF dimerization, DNA-binding and transcriptional activity and promoters of HIF degradation, with some inhibitors exhibiting more than one function. Inhibitors of HIF-1 mRNA and protein expression, include: lncRNA PIN1-v2 [46], S-TRPM2 calcium-permeable ion channel short variant [263] and EZN-2698 and EZN-2208 HIF-1α antisense oligonucleotides (ASOs) that inhibit HIF-1α mRNA and protein expression [264, 265]. The topoisomerase inhibitor, camptothecin analogue, topecan inhibits Hif-1α protein translation and function [266], the natural flavonoid Chrysin inhibits HIFα protein expression, by blocking Akt signaling [267], soybean glyceollin phytoalexins inhibit HIF-1α protein expression by blocking the PI3K/Akt/mTOR pathway [268], the estrogen metabolite 2-methoxy-estrodiol inhibits Hif-1α and Hif-2α protein synthesis, nuclear translocation and transcriptional activity and is currently under clinical evaluation in a variety of tumour types [269], and the small molecule inhibitor KC7F2 inhibits Hif-1α protein but not mRNA synthesis by repressing eukaryotic translation initiating factor 4E binding protein and p70 S6 kinase [270].

Inhibitors of Hif-1α accumulation and transcriptional activity, include the Hsp90 inhibitors GA, 17-AAG, 17DMAG and EC154 that promote VHL-dependent HIF-α degradation [271, 272], the HDAC inhibitor virinostat that promotes HIFα degradation [273], the small molecule PX12 that inhibits Hif-1α accumulation by targeting thioredoxin-1 [274, 275], the small molecular inhibitor LW6 that promotes VHL-dependent Hif-1α degradation [276] and BAY87–2243 that suppresses HIF-1α and Hif-2α protein accumulation by inhibiting mitochondrial complex-1 (stopped in phase 1 trials for safety reasons) [277, 278].

Inhibitors of HIF dimerization include cyclic CLLFVY that binds the HIF-1α PAS-B domain disrupting dimerization, transcriptional function and hypoxic response in tumour cells [279], TC-S7009 [280], an unreferenced small molecular PT2353 nanomolar HIF-2 but not HIF-1 inhibitor that impairs HIF DNA-binding and HIF-2 dependent hypoxic responses, and the antiseptic dye acriflavine that inhibits HIF-1 and HIF-2 and prevents HIF-1 dimerization [281]. The DNA intercalating anthracyclins Doxorubicin and daurubicin also inhibit HIF binding to HREs in gene promoters [282], and echinomycin (NSC-13502) prevents HIF-1 binding to the VEGF promoter core HRE sequence 5′-CGTG-3′ [283].

HIF transcriptional Inhibitors, include: chetomin dithio-diketopiperizine that impedes HIF-1α interaction with its transcriptional activating histone acetyltransferase p300 co-factor and increases the radiosensitivity of human fibrosarcoma cells [284]; idenopyrasole 21 that inhibits HIF-1 transcriptional activity but not HIF-1α accumulation or dimerization [285]; YC-1 platelet aggregation inhibitor that disassociates HIF-1α/p300 complexes, represses HIF transcriptional activity and reduces HIF-1α protein accumulation [286]; FM19G11 that inhibits HIF transcriptional activation by impairing interaction with p300 [287]; small molecule NSC-607097 that inhibits HIF-1 transcriptional activity [288], and IDF-11774 that prevents HIF-1α accumulation, regulates cancer metabolism, suppresses tumour growth in vitro and in vivo and is a clinical cancer therapy candidate [289]. Finally, the fungal product Chaetocin has been shown to de-regulate HIF-1α pre-mRNA splicing and inhibit hepatoma and ovarian cancer growth in cancer models by reducing angiogenesis (Fig. 6b) [290, 291].

#### Targeting the spliceosome

Targeting the spliceosome is an alternative way to inhibit tumour-promoting, hypoxia-induced alternative splicing.

Bacterial products that bind the SF3B component of U2 snRNP and interrupt spliceosome assembly, include: the spliceostatins, spliceostatin A, FD-895 and the derivatives FR901463, FR901464 and FR901465; sudemycins, from pseudomonas [292]; herboxidienes, from streptomyces A7847, and pladienolide B and its E7017 analogue, from streptomyces platensis Mer-11,107. These inhibitors induce cell cycle arrest, cytotoxicity and inhibit ≈10% of canonical splicing events, suggesting that only weaker splice sites are influenced by spliceosome inhibitors. E7017, spliceostatin A and sudemycin D6/K exhibit improved stability and lower inhibitory IC_50_ concentrations, making them more suitable for therapy [293–295]. The bi-flavonoid Isoginkgetin also inhibits splicing by preventing U4, U5 and U6 tri-snRNP recruitment to the spliceosome [296]. Recently, several novel inhibitors of the pre-mRNA splicing helicase Brr2, which orchestrates spliceosome rearrangements during splicing events [297], have been identified are currently being characterized (Fig. 6b) [298, 299].

#### RNA-based therapeutics for splice switching

RNA-based therapeutics have the potential to target any mRNA and, therefore, any protein, including proteins that lack catalytic activity, cannot be targeted by small molecular inhibitors or that are unamenable to antibody targeting.

Antisense oligonucleotides (ASOs) are the mainstay of RNA-based therapeutics. ASOs are 15-39mer chemically-modified RNA or DNA molecules that either redirect specific splicing events to prevent pathology-promoting splice isoforms or to generate isoforms that inhibit pathology. The first proof of concept for therapeutic ASOs efficacy in preventing aberrant alternative splicing has come from the FDA-approved ASO “Spiranza”, that targets survival motor neuron 2 (SMN2) pre-mRNA to promote exon 7 inclusion and full length SMN2 protein expression and has been successfully employed to treat spinal muscular atrophy [300, 301]. Target-specific ASOs can also be used to switch splicing by targeting 3′ or 5′ splice sites blocking their use, or to promote exon or intron inclusion by targeting splicing enhancer or silencer sequences. Unmodified DNA and RNA oligonucleotides are unstable and vulnerable to nuclease attack. Therefore, therapeutic ASOs contain chemical modified phosphate or ribose backbones, increasing stability and specificity, whilst maintaining low toxicity and immunogenicity and can also be used to induce RNAse H-mediated nonsense mediated mRNA decay. Common modifications include phospho-thioate linked backbones or 2′ O-(2-methoxyl) or 2′ O-methyl ribose modifications that increase half-lives from 2 weeks to 6 months, facilitating the use of shorter locked nucleic acid sequences with increased specificity and reduced off-target hybridizations. Phosphorodiamidate linkages in morpholino oligonucleotides further increases specificity and lowers toxicity but these ASOs must be conjugated with a delivery moiety for in vivo targeting [302]. Peptide nucleic acids ASOs are also highly specific, have been used to inhibit splicing events, are considerably more stable but exhibit lower solubility, limiting their use [303]. ASO delivery is also important and is achieved either systemically or by direct injection at site, with conjugation used to facilitate targeting (e.g. ASO conjugation with N-acetylgalactosamine promotes uptake by hepatocytes). In vitro conjugated ASOs that promote exon 3 inclusion in STAT3 by shifting axon 3a inclusion to exon 3b, which lacks nucleotides encoding the carboxyl terminal transactivation domain, induce apoptosis and tumour regression in a murine breast cancer model and targeted ASOs that induce MDM4 exon 6 skipping and decrease MDM4 protein levels reduce tumour growth in patient-derived xenograft melanoma and lymphoma models and are currently under clinical evaluation (Fig. 6b [304].

#### Chemotherapeutic agents that modify alternative splicing

Camptothecin and topotecan topoisomerase inhibitors, doxorubicin and cisplatin have all been reported to induce splicing changes in genes involved in DNA damage-repair, genetic stability and immortality [305, 306]. Doxorubicin also induces alternative splicing of the NF-YA component of the heterotrimeric ubiquitous transcription factor NF-Y in human neuroblastoma cells, resulting in expression of a cytotoxic NF-YAx exon 3 and exon 5 skipped isoform that, upon overexpression, induces neuroblastoma cell autophagic necroptosis [92]. Combinations of cancer drugs with splicing modulators have also been shown to enhance therapeutic efficacy, e.g. amiloride potentiates imatinib efficacy in chronic myeloid leukemia [307, 308] and sudemycin enhances ibrutinib efficacy in chronic lymphocytic leukemia (Fig. 6b) [309].

#### Targeting splicing factors

Splicing factors are divided into the serine/arginine-containing SR proteins: SRSFs 1–12, SC35, SRp20, SRp30c, SRp38, SRp40, SRp54, SRp55, SRp75, HTra2α, HTra2β1, 9G8, SF2/ASF and SRm160; nhRNPs: hnRNPA0, A1, A2/B1, A3, C, C1, C2, D, D0, DL, E1, E2, F, G, H1, H2, I (PTB), J, K, L, LL, M, Q, U, nPTB; and others: RBFox-1, RBFox-2,, DAZAP1, PSF, TDP43, RBM4, RBM5, RBM 10, RBM25, CUG-BP1, ESRP1, ESRP2, ETR-3, HuB, HuC, HuD, HuR, TIA-1, TIAL1, QK1, Sam68, SLM-1, SLM-2, SF1, SRSF2, SRSF3B1, U2AF1, FMRP, Nova-1, Nova-2, PRPF40B, KSRP, ZRANB2, MBNL1, YB-1, SAP155 ZRSR2 [310]. Splicing factors bind cis-ESE, ESS, ISE and ISS elements and recruit or interact with proteins that interact with RNA recognition motifs involved in sequence-specific RNA binding interactions and mRNA transport. Small molecule inhibitors can interfer with tertiary RNA structures, protein/RNA binding interactions and SR splicing factor binding to *cis enhancer or silencer elements* in introns and exons (e.g Spiranza which binds SMN2 mRNA to promote exon 7 inclusion by inhibiting an ISS) [300, 301]. Furthermore, hnRNPs are essential for normal eukaryotic cell function, survival, tumourigenesis [311, 312] and are therefore important potential therapeutic targets.

The oral selective inhibitor of nuclear export KPT-330 (Selinexor) [313], impairs hnRNP K and A1 nuclear-cytoplasmic shuttling in myelodysplastic syndrome and acute myeloid leukemia cells, providing a way to preferentially kill leukemia cells and exhibits encouraging anti-tumour activity in hematological and solid tumours [313, 314]. The Quinilone derivative 1-(4-methoxyphenyl)-3_(4-morpholino-6-nitroquinolin-2-yl)prop-2-en-1-one (compound 25) binds hnRNP K at micromolar concentrations, down-regulates c-Myc transcription and inhibits human cancer cell proliferation and human xenograft tumour growth in mice [315]. SiRNA knockdown of HnRNP A2/B1, which regulates pre-mRNA processing, mRNA metabolism, transportation and is implicated in various cancers, including advanced stage human gliomas, induces apoptosis and Ros generation and reduces the viability, adhesion, migration, invasion, chemoresistance of glioma cell lines (U251 and SHG44), identifying HnRNP A2/B1 as a relevant therapeutic target in gliomas [316]. HnRNPB1 expression correlates with lung cancer development and siRNA HnRNPB1 knockdown promotes A549 lung cancer cell apoptosis [317], and several potential inhibitory small HnRNPB1 binding molecules have also recently been identified amongst lung cancer drugs [318]. In contrast to full length HnRNP L splicing factor, the HnRNP L alternative exon 7 splice variant, which contains a stop codon, promotes head and neck squamous cell carcinogenesis and is therefore a potential target, and SRSF3 splice factor is also autoregulated by an alternative exon 4 splice variant in a manner similar to hnRNP L, and is promoted by hnRNP L. HnRNP L is also overexpressed in liver, lung and prostate cancer and siRNA HnRNP L knockdown inhibits prostate cancer cell proliferation and xenograft tumour growth in mice, and hnRNP L overexpression interacts with p53, cyclin p21 and Bcl2, identifying hnRNP L inhibition as a potential therapeutic strategy in prostate cancer (Fig. 6a) [319].

The ubiquitin proteasome pathway inhibitor Bortezomib reduces the proliferation CA46 and Daudi Burkitt lymphoma cells by down regulating the expression of high molecular weight sumoylated hnRNP K splicing factor and cMyc and up-regulating the expression of low molecular weight de-sumoylated hnRNP K, implicating sumoylated hnRNP K and cMyc repression in Bortezomib inhibition of Burkitt Lymphoma cell proliferation [320]. SiRNA hnRNPA1 knockdown inhibits HepG2 hepatocellular carcinoma cell proliferation, migration, promotes alternative PKM2 splicing and induces glycolysis, which influences glucose-dependent HnRNPA1 acetylation, de-acetylated under glucose starvation conditions by SIRT1 and SIRT6 sirtuins, which inhibit glycolysis by reducing PKM2 and increasing PMK1 expression, implicating an adaptive hnRNPA1 acetylation-regulated metabolic reprogramming mechanism for HCC metabolic adaptation, proliferation and tumourigenesis, within nutrient-deprived tumour microenvironments (Fig. 6a) [321].

#### Targeting splice factor kinases

Therapeutic targeting of splice factor kinases, which modulate splice factor involvement in spliceosome assembly, splice factor binding to splice sites and subsequently alternative splicing may also reduce the expression and activity of hypoxia-induced alternatively spliced tumour promoting protein isoforms [322]. Selective inhibitors of dual specificity CLK 1–4 splice factor kinases, activated by autocatalysis that phosphorylate SR proteins on serine/threonine residues to regulate alternative splicing, are being developed [322]. The small molecular nanomolar casein kinase-2 inhibitor CX-4945 inhibits CLKs1–4 and exhibits anti-proliferative, anti-angiogenic and anti-tumour activity in mouse tumor xenograft models, inhibits PI3K/Akt signaling and HIF1α transcription, and is currently in clinical trials for bile duct cholangiocarcinoma, with gemcetibine and cisplatin [323]. Other small molecule CLK inhibitors include TG-003 and KH-CB19 but their clinical potential in regulating splicing requires careful evaluation due to off-target side effects. The small molecule Wnt signaling pathway inhibitor SM08502 also inhibits CLKs (CLK3 and DRYKs) and oral SM08502 administration exhibits anti-tumor effects in mouse GI tumour xenograft models, inhibits SRSF phosphorylation, induces DVL2, TCFJ, ERBB2 and LRP5 alternative intron retention splicing and NMD, implicating alternative splicing in Wnt pathway signaling [324]. SM08508 inhibition of CLK2, CLK 3 and Wnt signaling is likely to disrupt spliceosomes, resulting in unstable alternative intron-retained splice transcripts, subsequently degraded by NMD. SM08508 also induces apoptosis regardless of the K-Ras or Wnt mutational status, permeates the nucleus and is in phase I clinical trials (NCT03355066) in patients with advanced stage solid tumours [324]. Amiloride, discovered in a screen of small molecule inhibitors of hepatocellular carcinoma Huh-7 cells, modulates oncogenic alternative splicing, devitalizes cancer cells, normalizes Bclx, HPK3 and RON/MISTR1 transcripts in association with SF2/ASF hypo-phosphorylation, reduces the expression of SRp20 and 2 other SR proteins, decreases AKT, ERK1/2 and PP1 phosphorylation and increases p38 and JNK phosphorylation, in association with global changes in alternative splicing, involving 584 exons in 551 ion transport, cell matrix formation, cytoskeletal remodeling and genome maintenance gene transcripts, reducing cellular invasion and migration, cell cycle disruption, cytokinesis and inducing cell death. Similar effects were also observed in myeloid leukemia and glioblastoma cells, identifying Amiloride as a novel small molecule modulator of oncogenic alternative splicing of therapeutic relevance (Fig. 6b) [325].

#### Targeting specific tumour promoting hypoxia-induced alternative spliced protein isoforms

Targeted therapies for the hypoxia-induced alternatively spliced EGFRvIII driver oncogene, include 1st generation (Gefitinib, Erlotinib and Lapatinib), 2nd generation (Afatinib and Dacomitinib) and 3rd generation (Rociletinib and AZD9291) tyrosine kinase inhibitors; EGFR L2 domain targeting antibodies (Cetuximab, Panitumumab and Nimotuzumab), the EGFRvIII-specific antibody mAB806 or antibodies conjugated with a toxins or radioisotopes (125ImAB425) and the double specificity (bis) antibody bscEGFRvIIIxCD3. A vaccine against EGFRvIII (Rindopepimut CDX110), however, has failed in phase III trials. CAR-T cells that target EGFRvIII are another promising option and are in phase I clinical trials, and RNA-based anti-EGFRvIII therapeutics are being evaluated in animal models [326, 327]. Therapeutic strategies for the inhibition of the hypoxia-promoted TrkAIII oncogene, a potential driver oncogene in cancers including neuroblastoma and Merkel cell carcinoma, include the small molecule Trk tyrosine kinase inhibitors, Larotrectinib, Entrectinib, Cabozantinib, Merestinib, TRS-011, DS-6051B, MGCD156, PLX486 and DCC-2710, FDA-approved or in clinical trials for cancers with Trk-fusion oncogenes or with altered Trk activity, that could be repurposed for treating tumours that express TrkAIII (Fig. 6b) [328]. Alternative approaches include TrkAIII-specific PNA inhibitors or equivalent siRNAs to repress TrkAIII expression, geldanamycin analogues, SOD-2 inhibitors, TRAIL or agents that target TrkAIII downstream signaling, such as PI3K/Akt/NF-kB inhibitors, the UPR, Bcl2, Bcl-xL and Mcl-1 proteins [90]. Recently, a novel humanized anti-RON antibody-drug conjugate Zt/g4-MMAE has been developed and validated for evaluation in the treatment of pancreatic cancer (Fig. 6b) [329]. Alternative approaches include: vaccination against tumour-specific splice variants with splice variant antigenic epitopes conjugated to keyhole limpet hemocyanin [330], the generation of CAR-T cells that target tumour-specific splice isoform [331] and the use of RNA and DNA ASOs, PNA, RNAi, ribozymes and adjuvant microRNA based strategies to reduce splice variant expression (see above).

## Conclusions

It is clear that tumour hypoxia-induced alternative splicing plays a fundamental role in all 10 cancer hallmarks, from initiation to metastatic growth, and is also a critical determinant of therapeutic resistance. The influence of hypoxia upon the basic mechanisms of pre-mRNA splicing including spliceosome assembly, splicing factor expression, activity and intracellular localization, miRNA synthesis and maturation, and the added influence of mRNA structure and elongation rate, results in an impressive number of alternatively spliced protein isoforms that promote tumor pathogenesis, metastatic progression and therapeutic resistance, warranting classification of hypoxia-induced alternative splicing as the 11th hallmark of cancer. Tumour hypoxia-induced alternative splicing provides a plethora of biomarkers of prognostic potential and therapeutic targets with potential to slow tumour progression and enhance therapeutic efficacy.

## Data Availability

Data supporting the conclusions of this article and all other data concerning reviewed articles, for which data was obtained at the University of L’Aquila, are available from the authors upon reasonable request.

## Abbreviations

HIF: Hypoxia-inducible factor
CREB: cAMP response element binding protein
STAT-3: Signal transducer and activator of transcription-3
VEGF: Vascular endothelila cell growth factor
VEGFR: Vascular endothelial cell growth factor receptor
TrkA: Tropomyosin-related kinase receptor A
EGFR: Epithelila growth factor receptor
TGF: Transforming growth factor
MARS: Methionine tRNA synthetase
CLK: CDC-like kinase
STPK: Serine/threonine protein kinase
MALAT: Metastasis-associated lung adenocarcinoma transcript
LUCAT: Lung cancer associated transcript
HOTAIR: HOT transcript antisense RNA
TNF: Tumour necrosis factor
Bcl: B-cell lymphoma
PI3K: Phosphoinositol-3-kinase
CK: Casein kinase
GW: Gawky bodies
PB: Processing bodies
NF-Y: Nuclear factor-Y
PTP: Protein tyrosine phosphatase
RON: Recepteur d’Origine Nantais kinase
MAPK: Mitogen-activated kinase
XBP: X-box-binding protein
AP-1: Activator protein-1
NF-kB: Nuclear factor kappa binding
mTOR: Mechanistic target of rapamycin kinase
ATR: Ataxia telangectasia and RAD3-related protein
BRCA: Breast cancer gene
BAX: Bcl-2 associated X
HDAC: Histone deacetylase
TP53BP1: Tumour protein p53 binding protein-1
TP53: Tumour promoting protein 53
Waf-1: Wild-type p53 activated protein
PTBP1: Polypyrimidine tract-binding protein-1
TERT: Telomerase reverse transcriptase
PHD: Hif prolyl hydroxylase
PDHC: Pyruvate dehydrogenase complex
PK: Pyruvate kinase
ARNT: Aryl hydrocarbon receptor nuclear translocator
EMMPRIN: Extracellular matrix metalloproteinase inducer
MiRNA: Micro RNA
EGFL: Epithelial growth factor-like
PGC-1a: Peroxisome proliferator-activated receptor coactivator 1-alpha
sVEGFR: Soluble vascular endothelila cell growth factor receptor
NK: Natural killer
TRAIL: TNF-related apoptosis inducing ligand
BNIP3: Bcl2/adenovirus E1B protein interacting protein-3
PERK: Pancreatic E1F2-alpha kinase
ER: Endoplasmic reticulum
UPR: Unfolded protein response
RIPK: Receptor-interacting serine/threonine kinase
HLA: Human leukocyte antigen
PD-1: Programmed death − 1
PD-L1: Programmed death-ligand 1
TDP-43: Tar DNA binding protein
MCP: Membrane cofactor protein
LDHA: Lactate dehydrogenase A
NMD: Nonsense-mediated RNA decay
EMT: Epithelila to mesenchymal transition
ZEB: Zinc finger E-box-binding homeobox
MFAP: Microfibrillar-associated protein
PTK: Protein tyrosine kinase
TRPM2: Transient receptor potential cation channel subfamily M member 2
GA: Geldanamycin
17-DMAG: 17-dimethylaminoethylamino-17-demethoxygeldanamycin
snRNP: Small nuclear ribonucleoprotein
hnRNP: Heterogeneous nuclear ribonucleoprotein
ASO: Antisense oligonucleotide
SMA: Spinal muscular atrophy
MDM4: Mouse double minute
Wnt-1: Wingless and Int-1
HPK: Homeodomain-interacting protein kinase
CAR-T: Chimeric antigen receptor-T
PNA: Peptide nucleic acid
siRNA: Small interfering RNA.
